# Functional characterization of missense variants affecting the extracellular domains of ABCA1 using a fluorescence-based assay

**DOI:** 10.1016/j.jlr.2023.100482

**Published:** 2023-12-03

**Authors:** Marianne Teigen, Åsa Schawlann Ølnes, Katrine Bjune, Trond P. Leren, Martin Prøven Bogsrud, Thea Bismo Strøm

**Affiliations:** Unit for Cardiac and Cardiovascular Genetics, Department of Medical Genetics, Oslo University Hospital, Oslo, Norway

**Keywords:** HEK293 cells, Tangier disease, cholesterol efflux, BODIPY-cholesterol, HDL, ApoA1, lipid metabolism, lipoproteins

## Abstract

Excess cholesterol originating from nonhepatic tissues is transported within HDL particles to the liver for metabolism and excretion. Cholesterol efflux is initiated by lipid-free or lipid-poor apolipoprotein A1 interacting with the transmembrane protein ABCA1, a key player in cholesterol homeostasis. Defective ABCA1 results in reduced serum levels of HDL cholesterol, deposition of cholesterol in arteries, and an increased risk of early onset CVD. Over 300 genetic variants in *ABCA1* have been reported, many of which are associated with reduced HDL cholesterol levels. Only a few of these have been functionally characterized. In this study, we have analyzed 51 previously unclassified missense variants affecting the extracellular domains of ABCA1 using a sensitive, easy, and low-cost fluorescence-based assay. Among these, only 12 variants showed a distinct loss-of-function phenotype, asserting their direct association with severe HDL disorders. These findings emphasize the crucial role of functional characterization of genetic variants in pathogenicity assessment and precision medicine. The functional rescue of ABCA1 loss-of-function variants through proteasomal inhibition or by the use of the chemical chaperone 4-phenylbutyric acid was genotype specific. Genotype-specific responses were also observed for the ability of apolipoprotein A1 to stabilize the different ABCA1 variants. In view of personalized medicine, this could potentially form the basis for novel therapeutic strategies.

CVD stands as the primary cause of morbidity and mortality worldwide (1). Low levels of HDL are considered a risk factor for CVD primarily because of its role in reverse cholesterol transport, a process wherein cholesterol from peripheral cells is transported by HDL to the liver for further metabolism or excretion (2, 3, 4, 5, 6). Nascent HDL particles are formed when cellular phospholipids and cholesterol are transported to lipid-free or lipid-poor apolipoprotein A1 (ApoA1) through the interaction with the cell surface ABCA1 (7).

ABCA1 is a member of the ABC transporter superfamily of proteins mediating trafficking of substrates across cell membranes and is highly expressed in the liver, brain, intestine, and macrophages (7, 8, 9). Comprising 2,261 residues, ABCA1 features two extracellular domains (ECD1 and ECD2) and two distinct transmembrane domains (TMDs) with six membrane-spanning helices each. Two intracellular nucleotide-binding domains follow, culminating in intracellular regulatory units (R). One R unit bridges the TMDs, whereas the other constitutes the C-terminal tail (10). A proposed model for the cholesterol efflux mechanism of ABCA1 entails ATP-dependent lipid translocation to membrane diffusive monomeric ABCA1 (11). The lipidation induces dimerization and immobilization of the ABCA1 dimer in the plasma membrane, whereby the transport of lipids to ApoA1 dissociates the dimer. In contrast to the lipid translocation mechanism described for the other members of the ABC transporter superfamily, ABCA1 was recently characterized as being an extracellular phospholipid translocase (12).

Genetic variants affecting ABCA1 functionality is evident in Tangier disease (OMIM #205400) caused by biallelic loss-of-function variants. Tangier disease manifests with absent or extremely low HDL cholesterol levels, accumulation of cholesterol deposits in extrahepatic tissues, and high risk of CVD (13). Familial hypoalphalipoproteinemia (OMIM #604091), caused by heterozygosity for loss-of-function variants in *ABCA1*, also faces an augmented risk of premature atherosclerotic CVD (14). As of June 2021, the Human Gene Mutation Database (HGMD) has cataloged over 300 variants in *ABCA1*. While many of these variants are clinically linked to dyslipidemia, only a few have undergone pathogenicity assessment by functional characterization. For functional characterization of ABCA1, cell culture-based cholesterol efflux analysis typically employs radioisotopes (15, 16, 17, 18, 19, 20, 21) or fluorescence-based methods (22, 23, 24). While radioisotope-labeled techniques have a higher sensitivity, their utility is limited by the need of specialized facilities, a low reagent shelf life, and radiation concerns. Conversely, fluorescence-based efflux assays are commercially available but can be economically prohibitive for large-scale screening initiatives.

In this study, we have refined a cost-effective and sensitive fluorescence-based assay allowing functional characterization of proteins involved in cholesterol efflux within standard laboratory conditions. Utilizing this approach, we have assessed the cholesterol efflux efficiency of missense variants affecting the ECDs of ABCA1. The evaluation of efflux efficiency, coupled with protein and in silico analyses, improves the accuracy of evaluating novel variants' pathogenicity and contributes to a broader understanding of the molecular pathways and metabolisms intrinsic to the human lipidome.

## Materials and Methods

### Numbering of nucleotides and codons of *ABCA1* and variant classification

Transcript NM_005502.4 was used for nucleotide and codon numbering of *ABCA1*. Codon number 1 was set to the ATG start codon and A of the start codon as nucleotide number 1. Classification of variant pathogenicity was based upon the guidelines from The American College of Medical Genetics and Genomics and The Association for Molecular Pathology (25). For clarification, the variants at protein level were annotated with the prefix p. throughout the article, except in the figure legends to reduce complexity.

### Study selection

This study encompasses all previously reported but not functionally characterized *ABCA1* missense variants within the sequence encoding the ECDs of ABCA1. These variants are located in ECD1, ECD2, and the connecting regions extending into the extracellular space amidst the transmembrane helices and extracellular helices 1 and 2 (EH1 and EH2), as outlined by Qian *et al.* (10). EH1 and EH2 might exhibit temporary localization between the extracellular space and the plasma membrane border. In total, this resulted in 45 variants from HGMD (www.hgmd.cf.ac.uk) up to June 2021. In addition, we included five novel *ABCA1* variants identified in hypoalphalipoproteinemic patients referred for genetic analyses to the Unit for Cardiac and Cardiovascular Genetics at Oslo University Hospital, Norway, along with one additional variant (p.Y793C) not reported in HGMD (26) (supplemental Table S1).

### Cell culture

ATP binding cassette subfamily G member 1 (ABCG1) is another member of the ABC transporter family, contributing to lipid transport to nascent HDL in synergy with ABCA1 (27). Therefore, human embryonic kidney 293 (HEK293) cells (European Collection of Authenticated Cell Cultures, Wiltshire, United Kingdom) with high transfection efficiency and minimal endogenous expression of *ABCA1* and *ABCG1* were used as the cellular model system. The HEK293 cells were cultured in HyClone MEM (GE Healthcare Life Sciences, Pittsburg, PA) supplemented with 10% fetal bovine serum, 2 mM l-glutamine (Sigma-Aldrich, St Louis, MO), 50 U/ml penicillin, 50 μg/ml streptomycin (GE Healthcare Life Sciences), and nonessential amino acids (Biowest, Nuaillé, France).

### Plasmids and transfections

A plasmid containing WT human *ABCA1* with a C-terminal FLAG epitope tag (pcDNA3.1-WT-*ABCA1*-FLAG) (17) was a generous gift from K. Hirano, Osaka University, Osaka, Japan. Three amino acid substitutions were present in this construct compared with transcript NM_005502.4 (Ensembl Genome Browser, release 109, https://ensembl.org/), namely p.R219K (c.969A>G), p.I883M (c.2962A>G), and p.K1587R (c.5073A>G). The latter substitution was described as being on the most common allele according to The Genome Aggregation Database (https://gnomad.broadinstitute.org/). The other two were mutagenized to Arg219 and Ile883, which were described as being on the most common alleles. A V5/His tag was amplified from pcDNA3.1/V5-His C (Invitrogen, Carlsbad, CA) adding flanking ApaI restriction sites. The pcDNA3.1-WT-*ABCA1*-V5/his (WT *ABCA1*) construct was obtained by restriction digestion and ligation utilizing an existing ApaI restriction site downstream of the *ABCA1* insert in pcDNA3.1-WT-*ABCA1*-FLAG. The WT *ABCA1* construct was used as a template to generate the different variants using QuickChange II XL Mutagenesis Kit (Agilent Technologies, Santa Clara, CA) according to the manufacturer’s instructions. As a negative control, the synthetic variant p.K939M (c.2816A>T) known to hamper the ATPase activity of ABCA1 was constructed (28). Furthermore, to assess the loss-of-function phenotype, we employed clinically and functionally validated Tangier disease-causing variants: p.R587W (c.1759C>T) (20, 29) and p.W590S (c.1769G>C) (19, 20), situated in ECD1, p.C1477R (c.4429T>C) (20, 30) in ECD2, and p.Y1767D (c.5299T>G) (31) in TMD2. The integrity of the plasmids was confirmed by DNA sequencing of the complete *ABCA1* open reading frame using primers with sequence overlap. The oligonucleotide sequences used for cloning, mutagenesis, and sequencing are listed in supplemental Table S2.

HEK293 cells were transiently transfected using FuGENE HD (Roche Diagnostics GmbH, Mannheim, Germany) in a 3:1 ratio of the amount of DNA for 24 h according to the manufacturer’s instructions. The general transfection efficiency of the plasmids was assessed by measuring mRNA levels using quantitative PCR (supplemental Fig. S1A). Intra-assay transfection efficiencies were monitored by co-transfection with a renilla luciferase plasmid, phRL (Promega, Madison, WI) at a ratio of 9:1 (supplemental Fig. S1B). The luminescence was measured using Renilla Luciferase Assay System (Promega) according to the manufacturer’s instructions and read on a Synergy H1 Plate Reader (BioTek, Winooski, VT).

### Isolation of HDL

Plasma was obtained from healthy blood donors, and HDL (ρ = 1.080–1.210 g/ml) was isolated by ultracentrifugation using an Optima XPN-80 ultracentrifuge and a type 70Ti fixed-angel rotor (Beckman Coulter Inc, Fullerton, CA). In brief, the plasma density (ρ = 1.006 g/ml) was adjusted to ρ = 1.063 g/ml using natrium bromide (Sigma-Aldrich), and centrifuged at 45,000 RPM (208,400 RCF) for 24 h at 10°C. The top layer consisting of non-relevant lipoproteins was discarded, and the remaining solution was adjusted to ρ = 1.210 g/ml prior to an additional centrifugation (45,000 RPM, 24 h, 10°C). The resulting top layer HDL fraction was dialyzed against PBS for removal of natrium bromide and kept in an argon atmosphere to prevent oxidation. The lipid profile and protein concentration of the HDL preparation were measured at the Department of Medical Biochemistry, Oslo University Hospital using standard methods. An ApoA1 concentration of >5.0 g/l in the HDL fraction was accepted.

### Cholesterol efflux assay

The cholesterol efflux assay was adapted from and optimized based on methods published by other groups (24, 32, 33). Briefly, HEK293 cells were seeded out at 30% confluence and transiently transfected the following day. After 24 h, the cells were washed with PBS at room temperature and loaded with BODIPY-cholesterol (Cayman Chemicals, Ann Arbor, MI) for 1 h at 37°C using MEM with 0.0045 mM BODIPY-cholesterol, 0.018 mM cholesterol, 5 mM Hepes, 2.5 mM methyl-β-cyclodextrin, 2% fatty acid-free BSA, and 1% fetal bovine serum (Sigma-Aldrich). One major refinement of the method from Sankaranarayanan *et al.* (24) was to utilize the cholesterol-solubilizing method from Widenmaier *et al.* (32). Briefly, BODIPY-cholesterol was dissolved in ethanol (0.45 mM) together with cholesterol (1.8 mM) and diluted to 0.009 mM in MEM with 10 mM Hepes and 5 mM methyl-β-cyclodextrin to keep the BODIPY-cholesterol in solution. The BODIPY solution was then mixed 1:1 with MEM containing 4% fatty acid-free BSA and 2% fetal bovine serum, making the final loading media. After cholesterol loading, the cells were washed twice with PBS and equilibrated for 12–16 h at 37°C in MEM without phenol red (Gibco Life Technologies, Paisley, UK) supplemented with 1 μg/ml avasimibe (Selleck Chemicals LLC, Houston, TX). To minimize cholesterol leakage in the equilibrium media, the BSA supplement reported in previous protocols (24, 33) was omitted. The cells were washed in PBS and incubated in acceptor medium consisting of MEM without phenol red with 100 μg/ml HDL for 4 h at 37°C. The acceptor medium was harvested and precleared by centrifugation to remove cell debris. The cell lysate was harvested by scraping in lysis buffer (1% Triton X-100 (Sigma-Aldrich), 150 mM NaCl, and 10 mM Tris-HCl [pH 7.4]) containing Complete™ Protease Inhibitor Cocktail (Roche Diagnostics) and lysed by sonication (40% amplitude, 0.5 cycle for 10 pulses) before removing cell debris by centrifugation. The amounts of BODIPY-cholesterol in 100 μl acceptor media and 10 μg of lysate were quantified on a Synergy H1 Plate Reader with an excitation and emission wavelength of 480 nm and 508 nm, respectively. Cholesterol efflux was calculated using the formula (medium signal/[medium signal + lysate signal]). Acceptor-unrelated cholesterol leakage was corrected for by subtracting efflux signal from mock-transfected cells without acceptor. ABCA1-unspecific cholesterol efflux was corrected for by subtracting efflux from mock-transfected cells from all *ABCA1* variant-expressing cells. Optimization and validation are presented in supplemental Figs. S2–S6. A baseline threshold for disease-causing variants was set to the cholesterol efflux of the control construct (p.W590S) with the highest activity (50% of WT ABCA1).

### Western blot analyses

HEK293 cells were harvested by scraping in lysis buffer and lysed by sonication or by incubation at −80°C for 30 min prior to cell debris being removed by centrifugation. A BCA Protein Assay kit (Pierce Biotechnology, Waltham, MA) was used to measure the protein concentration in the lysate supernatants according to the manufacturer's instruction. SDS-PAGE of 15 μg lysate was performed using 4–20% Criterion™ TGX™ Precast Gels (Bio-Rad, Hercules, CA), which were subsequently blotted to Immuno-Blot polyvinylidene difluoride membranes (Bio-Rad). Before immunostaining, the membranes were incubated with 5% Blotting Grade Blocker Non-Fat Dry Milk (Bio-Rad) for 1 h at room temperature to inhibit non-specific binding of antibodies. The amount of ABCA1 was analyzed using a HRP-conjugated anti-V5 antibody (R961-25; Invitrogen) binding to the C-terminal V5 tag. Two ABCA1-specific bands were often detected in the lysate, as seen by others (34, 35, 36). Both bands were quantified together and normalized to β-actin on the same blot. β-actin was detected by an anti-β-actin antibody from Abcam (ab213262; Cambridge, UK).

### Assays for determining transport efficiency of ABCA1 variants

The amount of ABCA1 present at the cell surface was determined using cell-surface biotinylation and confocal laser-scanning microscopy. In brief, HEK293 cells transiently transfected with the *ABCA1* plasmids were washed in PBS and incubated in serum-free minimal medium (OptiMEM; Gibco Life Technologies) overnight. The cells were washed two times in ice-cold PBS supplemented with 1 mM MgCl_2_ and 0.1 mM CaCl_2_ prior to incubation with 1 mg/ml EZ-Link Sulfo-NHS-LC-Biotin (Thermo Scientific) in biotinylation buffer (154 mM NaCl, 10 mM Hepes [pH 7.6], 3 mM KCl, 1 mM MgCl_2_, 0.1 mM CaCl_2_, and 10 mM glucose) for 30 min on ice. After two more wash steps, the unbound biotin was quenched with 100 mM glycine in ice-cold PBS for 30 min on ice and washed two times prior to harvesting by scraping in lysis buffer. The lysate was immunoprecipitated with Dynabeads™ MyOne™ Streptavidin T1 (Invitrogen) and analyzed by Western blot analysis. For confocal laser-scanning microscopy analyses, transiently transfected HEK293 cells seeded out on glass bottom dishes were washed in PBS and fixated in 2% paraformaldehyde at room temperature for 20 min. The dishes were washed three times in Hanks’ balanced salt solution (Gibco Life Technologies) before staining the cell membrane for 10 min with 7.5 μg/ml wheat germ agglutinin (WGA) Alexa Fluor™ 647 Conjugate (Invitrogen). After permeabilization with 0.05% saponin for 30 min, ABCA1 was stained with an anti-V5 antibody (R960-25; Invitrogen) and visualized using an Alexa Fluor™ 488-conjugated secondary antibody (A11001; Invitrogen). Cell nuclei were stained using Hoechst 33342 trihydrochloride trihydrate (Invitrogen). The cells were analyzed with a Zeiss LSM 700 inverted confocal laser-scanning microscope (Carl Zeiss Microscopy GmbH, Jena, Germany) using a 63× objective, and colocalization was assessed by the use of the Pearson’s correlation coefficient (*R*) obtained from the Zeiss Zen microscopy image system.

### Statistical analyses

Data are presented as mean (± standard deviations) unless otherwise stated. Using Stata, version 17.0 (StataCorp LLC, College Station, TX), *P* values were calculated with a two-sample *t-*test assuming equal or unequal variance, depending on results from an *F*-test checking for equal variances. The selected tail of the distribution (one-tailed or two-tailed) is stated in the figure legends. A significance level of *P* < 0.05 was used.

## Results

### Cholesterol efflux activity

**Fig. 1:**
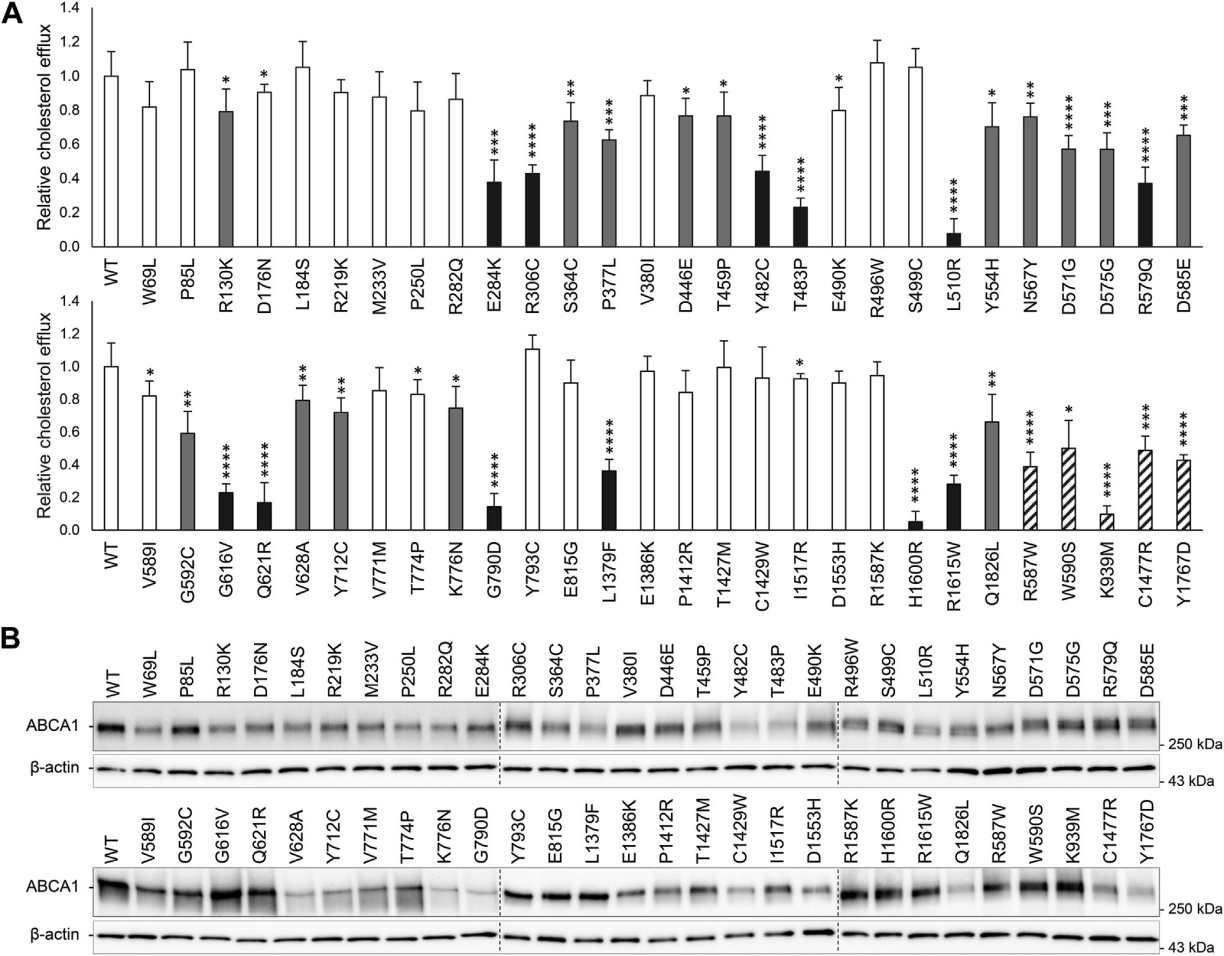
Cholesterol efflux and total ABCA1 protein. Functional analyses of HEK293 cells transiently transfected with 51 uncharacterized *ABCA1* variants and five previously characterized known loss-of-function control variants. A: Relative cholesterol efflux activity of benevolent variants (>80% efflux activity, white columns), loss-of-function variants (<50% efflux activity, black columns), variants of uncertain significance (50–80% efflux activity, gray columns), and loss-of-function control variants (black striped columns) normalized to WT ABCA1 (WT) of four independent experiments. Error bar represents 1 SD. ∗*P* < 0.05, ∗∗*P* < 0.01, ∗∗∗*P* < 0.001, ∗∗∗∗*P* < 0.0001, two-tailed *t-*test versus WT ABCA1. B: One representative Western blot showing total ABCA1 protein detected using a V5-HRP antibody. Relative total protein adjusted to level of β-actin is presented in supplemental Fig. S8. Dotted lines denote where blots have been merged.

**Fig. 2:**
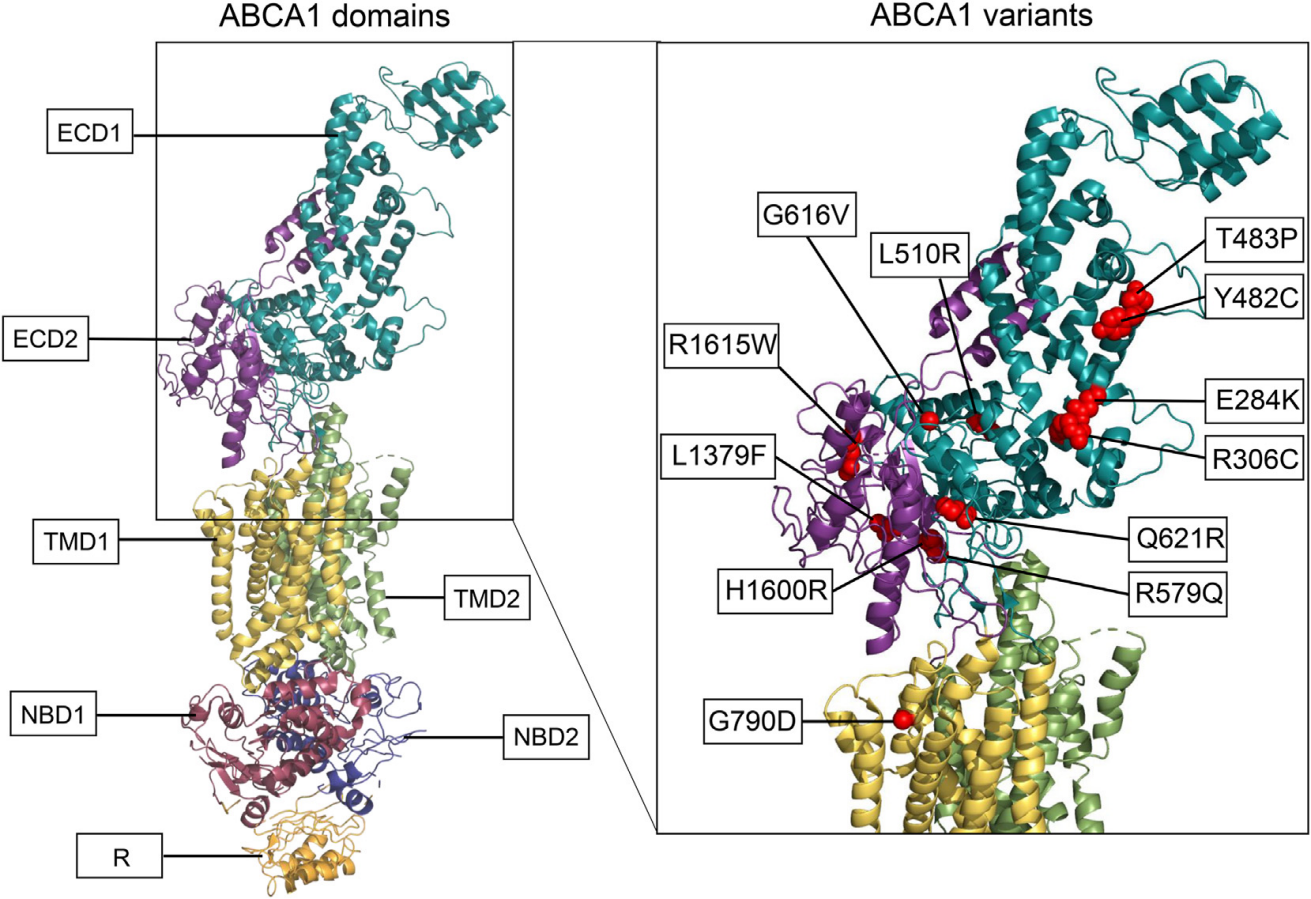
ABCA1 domain structure and localization of the 12 loss-of-function variants. The overall structure of ABCA1 colored by domain: two ECDs, ECD1 in cyan and ECD2 in magenta; two TMDs, TMD1 in yellow and TMD2 in green; two nucleotide-binding domains (NBDs) in red-violet and purple; and one regulatory (R) domain in orange. The enhanced picture on the right shows structural mapping of the 12 pathogenic variants in the ECDs and the loops of the TMDs. The structure is based on the Protein Data Bank structure 5XJY and made by the use of PyMOL (37).

The functionality of the 51 *ABCA1* variants was assessed by analyzing cholesterol efflux activity in transiently transfected HEK293 cells (Fig. 1A). Of the 51 analyzed variants, 12 (p.E284K, p.R306C, p.Y482C, p.T483P, p.L510R, p.R579Q, p.G616V, p.Q621R, p.G790D, p.L1379F, p.H1600R, and p.R1615W) demonstrated cholesterol efflux activities below the disease-causing threshold of 50% compared with WT ABCA1, classifying them as pathogenic loss-of-function variants (Fig. 1A). Of the 12 variants, 11 are localized in ECD1 or ECD2 (Fig. 2). The p.G790D variant is situated within an alpha helix in TMD1, thought to transiently extend into the extracellular space (10).

To establish a cholesterol efflux cutoff for variants assumed not to affect ABCA1 function, we observed that 10 of the tested variants (p.R130K, p.D176N, p.R219K, p.M233V, p.P250L, p.V380I, p.V589I, p.T774P, p.T1427M, and p.R1587K) appeared as reference sequences in more than two of the 29 species of vertebrates chosen for the multiple sequence alignment (supplemental Fig. S7). Given the presence of these variants in several species and the fact that these reference sequences are considered to encode functional proteins, we utilized the average of the three variants with the lowest cholesterol efflux activity (p.R130K, 79 ± 13%; p.P250L, 80 ± 17%; and p.V589I, 82 ± 9%) as the foundation for considering cholesterol efflux activity ≥80% as benevolent. Hence, variants exhibiting intermediate impact on cholesterol efflux activity (50–80% of WT ABCA1) present challenges in prediction without comprehensive clinical association data, designating them as variants of uncertain significance based solely on cholesterol efflux activities (Fig. 1A and supplemental Table S1).

### Effect of variants in *ABCA1* on the amount of ABCA1

The regulation of ABCA1 levels involves various cholesterol-dependent post-translational modifications, predominantly influencing the degradation rate. Furthermore, variants in *ABCA1* affecting protein folding and transport alter the overall degradation rate (38). To assess the impact of *ABCA1* variants on protein levels, we conducted Western blot analyses of HEK293 cells transiently transfected with each of the 51 variants (Fig. 1B). There was a significant difference in total ABCA1 protein levels in the majority of the variants compared with WT ABCA1 (supplemental Fig. S8). Of the 12 identified loss-of-function variants, 10 had significantly reduced ABCA1 levels, one (p.Q621R) had a similar amount as that of WT ABCA1, whereas one variant (p.G616V) displayed a significantly increased amount of ABCA1. Consequently, deviations in total protein levels may underlie deficient functionality but do not fully explain the differences in the cholesterol efflux caused by the different variants in *ABCA1*.

### Cell surface localization of ABCA1

**Fig. 3:**
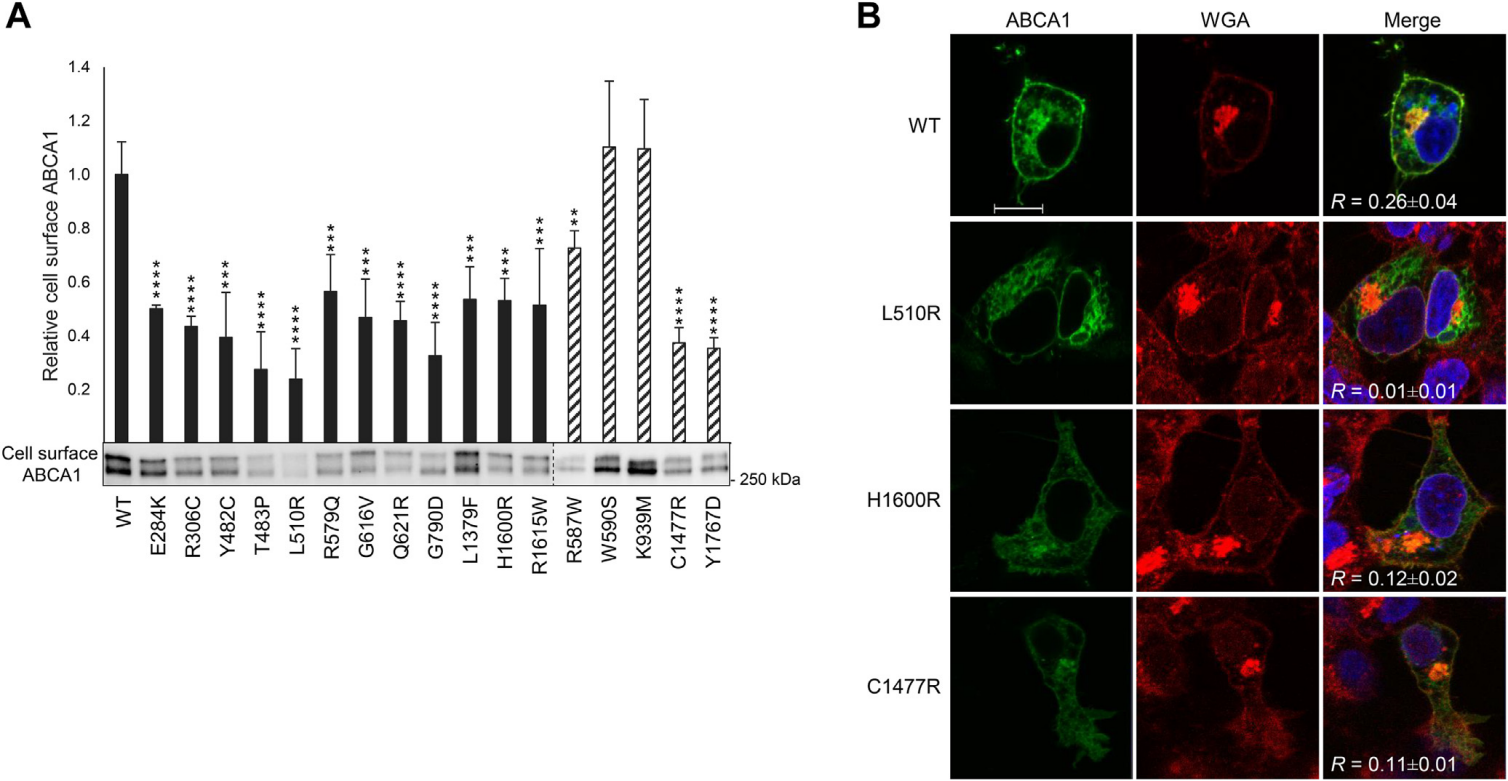
Cell surface expression of the 12 loss-of-function variants. Cell surface expression of ABCA1 was assessed in transiently transfected HEK293 cells. A: Relative cell surface expression, adjusted for total ABCA1 protein, of 12 loss-of-function variants (black columns) and five control variants (black striped columns) normalized to WT ABCA1 (WT) of four independent experiments. Error bar represents 1 SD. ∗∗*P* < 0.01, ∗∗∗*P* < 0.001, ∗∗∗∗*P* < 0.0001, two-tailed *t-*test versus WT ABCA1. One representative Western blot of immunoprecipitate is shown. The dotted line denotes where blots have been merged. B: Confocal laser-scanning microscopy of WT ABCA1 (WT), p.L510R (lowest surface expression in A), p.H1600R (intermediate surface expression in A), and control variant p.C1477R is shown. ABCA1 was detected using an anti-V5 antibody (green), and WGA (red) was used as membrane marker. Nuclei were stained with Hoechst (blue). Colocalization coefficient *R* is presented as mean ± SD of three independent experiments with at least three replicates per experiment. A scale bar of 10 μm is shown for WT ABCA1.

Certain genetic variants causing Tangier disease have previously been demonstrated to impair the translocation of ABCA1 to the cell membrane (19, 20). In addition, increased proteasomal degradation of misfolded proteins or proteolysis prompted by reduced interaction with ApoA1 can lead to reduced levels of mature ABCA1 at the cell surface, making it challenging to distinguish whether the phenotype results from increased degradation or impaired protein transport. Consequently, we subjected the 12 loss-of-function variants and the five control variants, including the established translocation-defective variant p.Y1767D (31), to biotinylation of surface-exposed ABCA1 (Fig. 3A). To mitigate discrepancies in protein levels arising from degradation, the amount of immunoprecipitated lysates was corrected for the total ABCA1 protein level quantified for each sample. All 12 loss-of-function variants exhibited reduced levels of biotinylated ABCA1 at the cell surface. Whereas, p.W590S and p.K939M exhibited surface expression levels comparable to that of WT ABCA1, the control p.C1477R demonstrated a similar degree of translocation deficiency as p.Y1767D.

To add to the protein analyses, the two variants with the most significant deficiency in cholesterol efflux activity (p.L510R and p.H1600R) and the control variant p.C1477R were each transiently transfected into HEK293 cells and examined by confocal laser-scanning microscopy (Fig. 3B). The WT ABCA1 protein demonstrated distinct membrane distribution and colocalization with the plasma membrane marker WGA (*R* = 0.26 ± 0.04). Whereas p.H1600R showed pronounced reduced cell-surface localization (*R* = 0.12 ± 0.02), the colocalization with WGA was negligible for p.L510R (*R* = 0.01 ± 0.01), consistent with the biotinylation data (Fig. 3A). The translocation deficiency of the control p.C1477R was also reproduced by a significantly reduced cell surface localization compared with that of WT ABCA1 (*R* = 0.11 ± 0.01).

### Functional rescue of ABCA1 loss-of-function variants

**Fig. 4:**
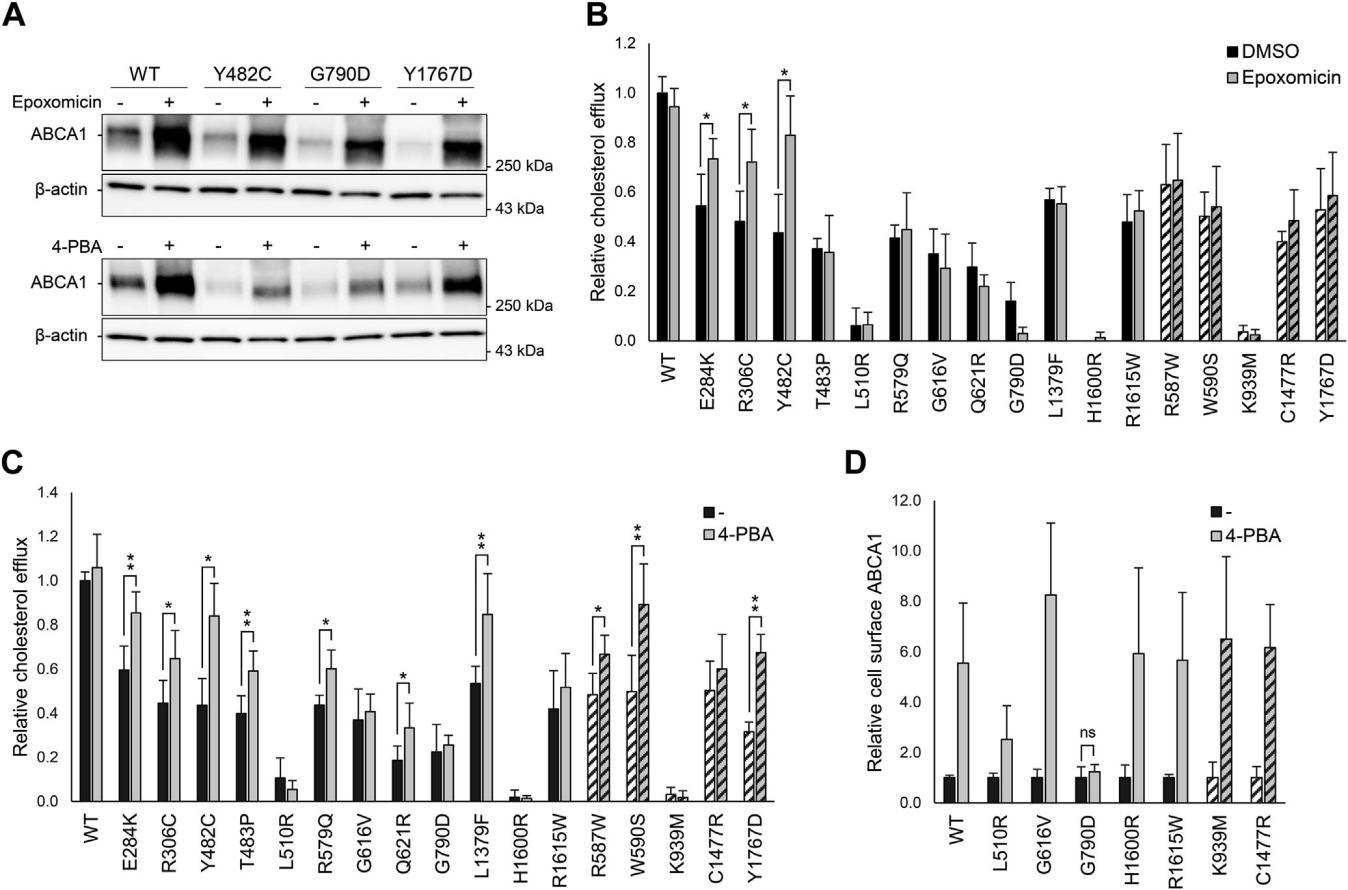
Functional rescue of ABCA1 loss-of-function variants. Rescue of ABCA1 functionality by 20 h incubation with epoxomicin (1 μM) or 4-PBA (10 mM) assessed for 12 loss-of-function variants and five control variants. A: Total ABCA1 protein from a selection of transiently transfected HEK293 cells incubated with epoxomicin or 4-PBA analyzed by Western blot analysis. One representative blot of three independent experiments is shown. Western blot and relative total ABCA1 protein corrected for β-actin for all 12 variants are found in supplemental Figs. S9A and S10A. Relative cholesterol efflux activity normalized to mock-treated WT ABCA1 (WT) presented as mean of three independent experiments for transiently transfected HEK293 cells incubated with (B) epoxomicin (gray columns) or equal volume of DMSO (black columns) or (C) 4-PBA (gray columns) or equal volume of dH_2_O (black columns). Error bar represents 1 SD (∗*P* < 0.05, ∗∗*P* < 0.01, one-tailed *t-*test vs mock-treated cells). Control variants are shown as striped columns. D: Relative cell surface expression analyzed by Western blot analysis of variants and controls not regaining functionality by 4-PBA treatment. The samples are normalized to mock-treated cells presented as mean of three independent experiments (*P* < 0.05, one-tailed *t-*test vs mock-treated cells). Controls are shown as striped columns. Error bar represents 1 SD. Nonsignificant expression difference (ns) is indicated. One representative Western blot is presented in supplemental Fig. S10B.

Given the reduced cell surface protein levels observed for all 12 loss-of-function variants, we explored the possibility for functional rescue of the mutated proteins. Proteasomal inhibition has been proposed as a therapeutic strategy targeting mislocalized or transport defective proteins in other pathologies, and degradation of ABCA1 has been shown to be strongly impaired by proteasomal inhibitors (38, 39). To this end, transiently transfected HEK293 cells were subjected to treatment with the proteasomal inhibitor epoxomicin (Sigma-Aldrich) for 20 h and analyzed for protein levels and cholesterol efflux (Fig. 4). The inhibition of proteasomal degradation led to an increase of total ABCA1 protein levels for all variants, including WT ABCA1, confirming that a portion of the ABCA1 protein normally undergoes proteasome-mediated degradation (Fig. 4A and supplemental Fig. S9A). However, the elevated expression levels only translated into a significant increase in cholesterol efflux for the three variants p.E284K, p.R306C, and p.Y482C (Fig. 4B). Despite a lack of functional rescue for the remaining nine variants, their cell surface levels were markedly increased (supplemental Fig. S9B). Notably, despite a 3-fold increase in cell surface expression, p.G790D exhibited a noticeable reduction in cholesterol efflux activity upon epoxomicin treatment (Fig. 4B).

The chemical chaperone 4-phenylbutyric acid (4-PBA) interacts with exposed hydrophobic segments and aids in the folding of proteins in the endoplasmic reticulum (ER), thereby mitigating ER stress (40). 4-PBA has been shown to be capable of restoring functionality in transport-defective ABCA1, including the Tangier disease-associated variant p.Y1767D (31). Consequently, HEK293 cells transiently transfected with each of the 12 loss-of-function variants were treated with 4-PBA (Sigma-Aldrich) for 20 h and analyzed for ABCA1 levels, cholesterol efflux, and cell surface expression (Fig. 4). Similar to epoxomicin, 4-PBA elevated ABCA1 levels for all variants (Fig. 4A and supplemental Fig. S10A). As seen with epoxomicin, functional rescue of cholesterol efflux was genotype dependent (Fig. 4C). The cholesterol efflux activity of the variants p.E284K, p.R306C, and p.Y482C was increased, which was also seen when inhibiting proteasomal degradation. Furthermore, p.T483P, p.R579Q, p.Q621R, and p.L1379F showed a marked boost in cholesterol efflux. While none of the pathogenic control variants responded to epoxomicin, p.R587W and p.W590S, in addition to p.Y1767D, regained some activity when treated with 4-PBA. Despite the absence of a functional response to 4-PBA, the variants p.L510R, p.G616V, p.H1600R, and p.R1615W exhibited a 3–8-fold increase in ABCA1 surface levels (Fig. 4D and supplemental Fig. S10B). The surface expression of p.G790D remained unresponsive to 4-PBA. Notably, there was no discernible discrepancy in the extent of ER stress between WT ABCA1 and the loss-of-function variants, implying that none of the variants led to detrimental accumulation of misfolded protein in the ER (supplemental Fig. S11).

### Stabilization of ABCA1 loss-of-function variants

**Fig. 5:**
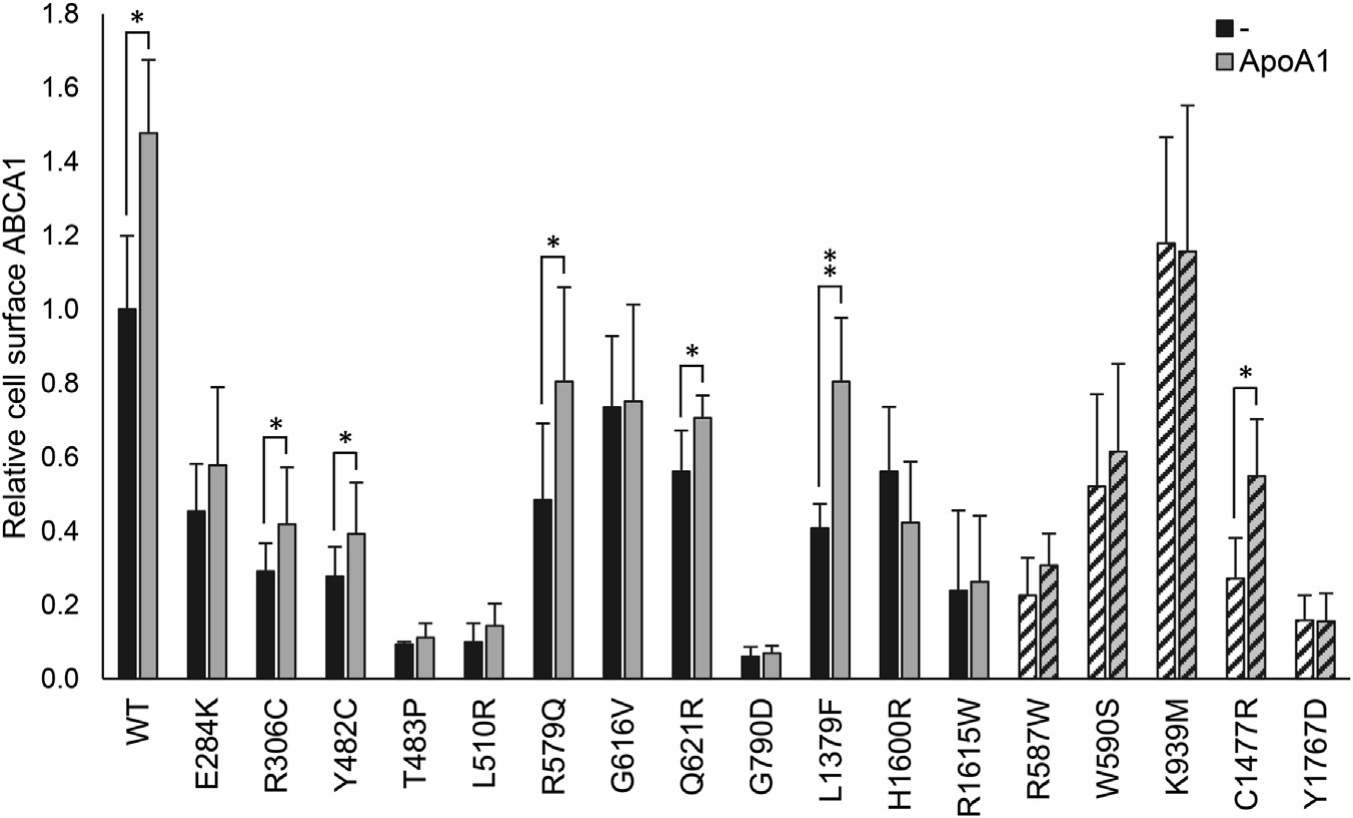
Stabilization of ABCA1 loss-of-function variants by recombinant ApoA1. Relative cell surface expression for the 12 loss-of-function variants (black columns) and five control variants (striped columns) normalized to WT ABCA1 (WT) after stabilization by 10 μg/ml recombinant ApoA1 (gray columns) for 2 h. The samples are presented as mean quantified protein of three independent Western blot experiments (∗*P* < 0.05, ∗∗*P* < 0.01, one-tailed *t-*test vs mock-treated cells). Error bar represents 1 SD. One representative Western blot is presented in supplemental Fig. S12.

ApoA1 plays a pivotal role in stabilizing and safeguarding the ABCA1 protein against calpain-mediated degradation at the plasma membrane (41). Given that all 12 loss-of-function variants exhibited compromised transport capabilities, a reduced ability to bind to ApoA1 at the cell surface will be expected. To circumvent the translocation deficiency, we examined the interaction between ABCA1 and ApoA1 by assessing the potential of ApoA1 to stabilize the mutated proteins at the cell surface (42). In this assay, HEK293 cells transiently transfected with each of the 12 loss-of-function variants were treated with 10 μg/ml recombinant human ApoA1 (Abcam) for 2 h at 37°C prior to biotinylation and subsequent analysis of cell surface ABCA1 levels (Fig. 5 and supplemental Fig. S12). While WT ABCA1 gained a 50% stabilization (*P* < 0.05), the negative control p.K939M displayed no responsiveness to ApoA1 treatment. In accordance with the functional rescue obtained for five of the variants (p.R306C, p.Y482C, p.R579Q, p.G621R, and p.L1379F) (Fig. 4), these variants were also significantly stabilized by recombinant ApoA1 indicating that these variants retain some ability to interact with the cholesterol acceptor. Conversely, the remaining seven variants exhibited negligible response to recombinant ApoA1, indicating diminished or defective interaction with the cholesterol acceptor. Adding to the discrepancy in the functional assessment of p.C1477R, this variant was significantly stabilized by ApoA1.

## Discussion

Although genetic variants causing minor reductions in HDL cholesterol levels are not associated with atherosclerotic coronary artery disease, clinical data connect monogenic HDL deficiency disorders, like those caused by *ABCA1* loss-of-function variants, to a heightened risk of early onset CVD (13). In this study, we have examined 51 *ABCA1* missense variants associated with HDL deficiencies, of which only 12 could be confirmed pathogenic by functional assays. Thus, evaluating the pathogenicity of genetic variants in *ABCA1* by functional characterization is of clinical importance. Whereas, functional characterization strengthens pathogenicity assessments, screening techniques must be accessible and cost effective for functional studies to be implemented. Herein, we have optimized a sensitive fluorescence-based cholesterol efflux assay suited for large-scale screening in a standard laboratory environment.

Focusing on variants affecting the ECDs of ABCA1, 33 variants were clustered in ECD1, 11 variants in ECD2, and seven variants were located in the hinges (EH) or protruding extracellular linkers between the transmembrane helices. In accordance with other studies (20), we observed a significant difference in ABCA1 levels in lysates between the majority of the variants studied, compared with that of the WT ABCA1. This indicates that even minor structural alterations could influence the overall ABCA1 protein level, although there was no clear translation to cholesterol efflux capability.

Previous categorization studies have grouped *ABCA1* variants into four types based on dysfunction (43): maturation-defective variants, lipid translocation-defective variants, ApoA1-binding defective variants, and variants rendering abnormal ATP hydrolysis. As the 12 loss-of-function variants identified in this study exhibited diminished protein levels at the cell surface, all these variants are assumed to be at least partly maturation defective. Since many of the loss-of-function variants had almost as much as 50% cell surface located protein compared with that of WT ABCA1, we questioned whether they also were defective in ApoA1 binding or lipid translocation.

From a personalized medicine perspective, understanding the specific protein dysfunction is essential for guiding therapeutic strategies, as evidenced by treatments for other disorders (44, 45). Approaches that target the ubiquitin-proteasome system or employ chemical chaperones may restore lipid transport functionality especially in patients with maturation-defective *ABCA1* loss-of-function variants (31, 38). Given existing reports on the polyubiquitination and proteasomal degradation of ABCA1 (38, 46), we explored the extent to which protein dysfunction could be rescued and bypassed by using the proteasome inhibitor epoxomicin and the chemical chaperone 4-PBA.

Whereas, epoxomicin is a specific inhibitor of the proteasome, 4-PBA interacts with the hydrophobic domains of misfolded proteins and increase the protein-folding capacity of the ER by reducing aggregation (47). Both epoxomicin and 4-PBA elevated the total protein level of all ABCA1 variants in our study. This suggests that a considerable amount of overexpressed ABCA1 is normally degraded through the ER-associated protein degradation pathways, corroborating the findings of Hsieh *et al.* (38). Furthermore, none of the variants caused increased cellular ER stress compared with that of the WT ABCA1. However, only three variants (p.E284K, p.R306C, and p.Y482C) regained functionality when epoxomicin was employed. Interestingly, in addition to increasing cholesterol efflux for these three variants, 4-PBA increased the functionality of variants not rescued by epoxomicin (p.T483P, p.R579Q, p.Q621R, and p.L1379F). This observation indicates that degradation induced by aggregation of misfolded proteins, rather than degradation through the ubiquitin-proteasome system, may be a more common cause of the ABCA1 loss-of-function phenotype (48). Whereas 4-PBA has been shown to restore both plasma membrane localization and cholesterol efflux function for other translocation-defective ABCA1 variants (31), the rescue effect was clearly dependent on the genotype.

Five variants (p.L510R, p.G616V, p.G790D, p.H1600R, and p.R1615W) did not exhibit restoration of functionality with epoxomicin or 4-PBA. To delve deeper into this issue, we examined the cell membrane localization of ABCA1 for these variants. Whereas epoxomicin treatment notably increased cell surface protein levels for all variants, the effect of 4-PBA was genotype dependent. There was a chaperone-induced increase in the level of surface-exposed ABCA1 for p.G616V, p.H1600R, and p.R1615W, and to a lesser extent for p.L510R. However, there was no observed increase in ABCA1 protein level on the cell surface for p.G790D.

The p.G790D variant is situated within an alpha helix in the TMD, which is thought to occasionally protrude into the extracellular space (10). A detailed examination of the Gly790 position within the ABCA1 protein reveals a region composed of closely packed alpha helices, which are most likely positioned within the hydrophobic lipid bilayer. Residue Gly790 is highly conserved across species, and the substitution of the small glycine with the larger and highly hydrophilic aspartic acid is likely to significantly impact protein folding, rendering the mutated protein dysfunctional and preventing it from reaching the cell surface. Accordingly, this variant exhibited one of the lowest levels of cholesterol efflux of all variants analyzed in this study. The p.G790D variant was identified in a male patient exhibiting symptoms of Tangier disease and a low HDL cholesterol level of 0.18 mmol/l (49). This patient was compound heterozygous for variants p.G790D and p.N1800H (c.5398A>C). The p.N1800H variant has previously been investigated by Sorrenson *et al.* (31), who found a 30% reduction in cholesterol efflux levels compared with that of WT ABCA1. Therefore, it remains uncertain whether the low HDL cholesterol level in this patient is primarily attributed to one of the two variants or a combination of both.

Whereas, 4-PBA restored plasma membrane localization for the four variants p.L510R, p.G616V, p.H1600R, and p.R1615W, they did not regain functionality. Given that variants in ECDs are unlikely to impair ATP hydrolysis, it is highly probable that these variants have defective lipid translocation or ApoA1-binding capabilities. Furthermore, ABCA1 variants causing impaired interaction with ApoA1 have been reported (19). To add to this notion, we examined if the 12 loss-of-function variants influenced the stabilizing effect that recombinant ApoA1 has on ABCA1 protein, as an indicator of preserved protein functionality (41). A subset of variants exhibited a significant upregulation of cell surface ABCA1 upon stabilization by ApoA1. In contrast, the variants p.T483P, p.L510R, p.G616V, p.G790D, p.H1600R, and p.R1615W remained unaffected by ApoA1 treatment. Five of these six non-responsive variants match those unresponsive to 4-PBA treatment, indicating that their dysfunction is not solely because of impaired transport to the cell surface. Interestingly, despite being categorized as an ApoA1 binding-defective variant (20, 50), p.C1477R exhibited enhanced stability when treated with recombinant ApoA1 in our study. We suspect that this variant may influence ABCA1 function in multiple ways. We postulate that the elevated levels of recombinant ApoA1 facilitate binding to the mutated low-affinity binding site in p.C1477R, thereby compensating for some of the functional deficits of the loss-of-function variant. In contrast to other reports, our findings also indicate that levels of cell surface p.C1477R are reduced compared with that of WT ABCA1, leading us to hypothesize that this variant may impede the maturation and/or transport of ABCA1 to the cell surface in addition to exhibiting defective ApoA1 binding. An ambiguity arises as both WT ABCA1 and the reported binding-deficient p.C1477R responded to ApoA1 stabilization. Therefore, the degree of stabilization falls short in confirming if the variant dysfunction is due to inadequate ApoA1 binding.

There is currently a phase 3 trial underway assessing the potential of recombinant ApoA1 in reconstituted HDL (CSL112) as a therapeutic strategy for preventing early recurrent cardiovascular events after a myocardial infarction (51). CSL112 has demonstrated a dose-dependent increase in cholesterol efflux, reaching up to a 4-fold increase in healthy adult subjects (52). Notably, its therapeutic efficacy for patients with variants in *ABCA1* would be contingent upon the ability of ABCA1 to interact with ApoA1 and that the variant has some retained functionality. Variants, such as p.L510R, p.G616V, p.G790D, p.H1600R, and p.R1615W, displayed little or no retained function, were not stabilized by ApoA1, and gained no resurgence in cholesterol efflux activity after treatments with epoxomicin or 4-PBA. Thus, our findings suggest that individuals with these variants might not benefit from recombinant ApoA1 infusion, emphasizing the significance of functionally characterizing genetic variants for personalized medicine.

In a recent benchmark study by Segrest *et al.* (12) ABCA1 is postulated to act as an extracellular phospholipid translocase, in contrast to the generally accepted alternating-access model for substrate export by ABC transporters (53). Segrest *et al.* identified two new central subdomains in ECD1, namely the gateway (residues 564–592) and annulus (residues 69, 71–80, 363, and 368–379) domains. As a translocase, ABCA1 extracts lipids from the outer face of the plasma membrane and forces phospholipids through the gateway and annulus into the elongated hydrophobic tunnel of ECD1. Salt-bridge formation between the charged residues of the gateway and the phospholipids are crucial for this translocation. Whereas none of the variants characterized in our study are located in the central portion of the annulus domain (residues 73–74, 77, 371, and 375), seven variants (p.N567Y, p.D571G, p.D575G, p.R579Q, p.D585E, p.V589I, and p.G592C) are located in the gateway domain. Of these, only p.V589I is characterized as a benevolent variant with normal functionality, which likely can be attributed to the similarities in size and properties between the amino acids valine and isoleucine. In contrast, p.R579Q was the only one of these seven variants that is characterized as loss of function, the other five variants have reduced functionality but with uncertain significance. Our findings align with the suggestion that the charged residues in the gateway domain appear to be important for the functionality of ABCA1. All the 12 loss-of-function variants characterized in our study presented with transport deficiency and reduced cell surface expression of ABCA1. Interestingly, none of the variants studied by Segrest *et al.* (12) deferred from the control construct in regard to cell surface expression. Although different cell model and overexpression systems are utilized, our results suggest the importance of considering ABCA1 protein turnover when assessing cell surface expression.

We found no direct association between amount of total ABCA1 protein and cholesterol efflux activity. This observation underscores that total ABCA1 protein levels may not accurately reflect functionality; instead, for certain variants, they may suggest protein aggregation and consequent dysfunction. Whereas 4-PBA and epoxomicin led to a notable elevation in both total and surface WT ABCA1 protein, this did not, however, translate to a corresponding increase in cholesterol efflux activity. This raises the question about the suitability of the overexpression system and assay employed in this study for examining potential *ABCA1* gain-of-function variants. It is plausible that *ABCA1* variants that generally enhance the cholesterol efflux activity of the transporter may not be advantageous for the cell, as they may disrupt cholesterol homeostasis and potentially influence the risk of cardiovascular events. Furthermore, given the substantial turnover rate of ABCA1 protein, one could speculate if a gain-of-function phenotype would be attenuated by increased degradation.

Evaluating available clinical data for the 12 loss-of-function variants showed a consensus with our findings, in which all the variants are being associated with HDL cholesterol levels in the lowest 10th percentile and decreased levels of ApoA1 (supplemental Table S3). However, this lipid profile is also reported for the majority of subjects harboring the other missense variants characterized in this study, many of whom are heterozygous. A large number of these variants were originally reported from screening studies of large patient cohorts, with the inclusion criteria being severely reduced serum HDL cholesterol levels. Taking into account the considerable impact polygenic and lifestyle factors have on serum lipid levels, and the large number of genetic variants reported in *ABCA1*, the clinical genotype-phenotype association is uncertain. In comparison, pathogenicity assessment and phenotype association are strongly asserted when emphasized by case reports on homozygous patients. During the finalization of this article, Barbosa-Gouveia *et al.* (54) reported the finding of p.H1600R in a clinically verified Tangier disease patient. The homozygous male patient had splenomegaly, hepatosplenomegaly, a HDL cholesterol level of 0.05 mmol/l, and no detectable ApoA1 in serum, in addition to a family history of premature cardiovascular events.

Through a combination of experimental techniques, we have ascertained the pathogenicity of 51 *ABCA1* missense variants, of which 12 were identified as being pathogenic loss-of-function variants. A notable proportion of the *ABCA1* variants studied aligned with predictions based on bioinformatic tools like PolyPhen-2 (55), MutationTaster (56), SIFT (57), and with AMCG/AMP guideline classification (25). However, some discrepancies between clinical presentations, functional characterizations, and in silico predictions emphasize, notably for the loss-of-function variants, the indispensability of functional tests in ascertaining the pathogenicity of novel genetic variants. Our results suggest potential genotype-dependent therapeutic strategies for HDL deficiency patients, including the infusion of recombinant ApoA1. Ultimately, understanding the underlying molecular mechanisms of the dyslipidemic phenotypes could pave the way for personalized treatment strategies in the future.

## Data availability

All data supporting the findings of this study are available within this article and its supplemental data.

## Supplemental data

This article contains supplemental data (12, 23, 24, 25, 26, 32, 33, 37, 54, 55, 56, 57, 58, 59, 60, 61, 62, 63, 64, 65, 66, 67, 68, 69, 70, 71, 72, 73, 74, 75, 76, 77, 78, 79, 80, 81, 82, 83, 84, 85, 86, 87, 88).

## Conflict of interest

The authors declare that they have no conflicts of interest with the contents of this article.
