# Supplementary material for: Functional characterization of missense variants affecting the extracellular domains of ABCA1 using a fluorescence-based assay
Source: J Lipid Res. 2023 Dec 3;65(1):100482. doi: 10.1016/j.jlr.2023.100482 (PMC10792246; doi:10.1016/j.jlr.2023.100482)
Supplement: Supplemental data [file mmc1.pdf]

Supplemental material

Functional characterization of missense variants affecting the extracellular domains of ABCA1 using a fluorescence-based assay

Marianne Teigen, Åsa Schawlann Ølnes, Katrine Bjune, Trond P. Leren, Martin Prøven Bogsrud and Thea Bismo Strøm

Supplemental Table S1: Missense variants in *ABCA1* subjected to functional characterization. Missense variants in *ABCA1* not previously functionally characterized are annotated with respect to their effects at the protein level and at the nucleotide level. The reported lipid associated phenotypic effects of the variants, as well as the classifications made by HGMD for variants reported in that database are shown. Also shown are the *in silico* predictions of pathogenicity generated by Sorting Intolerant from Tolerant (SIFT) [56], MutationTaster [55] and Polymorphism Phenotyping v2 (PolyPhen2) [54]. Allele frequencies of the variants are obtained from the Genome Aggregation Database (https://gnomad.broadinstitute.org/) (gnomAD), and pathogenicities of the variants assessed according to the ACMG guidelines [25] are shown.

Variant				*In silico* predictions					
Protein level	Nucleotide level	Phenotype*	HGMD class[#]	SIFT	Mutation Taster	PolyPhen2	gnomAD[&]	ACMG class[§]	Ref.
p.W69L	c.206G>T	Low HDL-C	DM?	Deleterious	Deleterious	Probably Damaging	-	3	[58]
p.P85L	c.254C>T	Low HDL-C	DM	Deleterious	Deleterious	Probably Damaging	1/203 (EF)	2	[59]
p.R130K	c.389G>A	Low HDL-C	DM?	Tolerated	Benign	Benign	-	2	[60]
p.D176N	c.526G>A	-	DM	Tolerated	Deleterious	Benign	1/3589 (EF)	2	[61]
p.L184S	c.551T>C	-	DM?	Deleterious	Benign	Benign	1/14213 (NFE)	2	[62]
p.R219K	c.656G>A	High HDL-C	FP	Tolerated	Benign	Benign	1/2 (A)	1	[63]
p.M233V	c.697A>G	-	DP	Tolerated	Benign	Benign	-	2	[64]
p.P250L	c.749C>T	-	DM	Tolerated	Deleterious	Benign	1/5025 (EF)	2	[61]
p.R282Q	c.845G>A	Low HDL-C	DM	Tolerated	Benign	Benign	1/8311 (A)	3	[65]
p.E284K	c.850G>A	Low HDL-C	DM	Tolerated	Deleterious	Benign	-	3	[66]
p.R306C	c.916C>T	Low HDL-C	DM?	Deleterious	Deleterious	Probably Damaging	1/16256 (A)	3	[67]
p.S364C	c.1091C>G	Low HDL-C	DM	Tolerated	Deleterious	Probably Damaging	1/113738 (NFE)	3	[68]
p.P377L	c.1130C>T	Tangier	DM	Deleterious	Deleterious	Probably Damaging	1/30616 (SA)	4	[69]
p.V380I	c.1138G>A	-	-	Tolerated	Benign	Benign	1/11813 (L)	2	Novel

p.D446E	c.1338C>G	-	DM?	Tolerated	Benign	Benign	1/157 (AJ)	2	[62]
p.T459P	c.1375A>C	Low HDL-C	DM?	Tolerated	Benign	Benign	-	3	[70]
p.Y482C	c.1445A>G	Low HDL-C	DM	Tolerated	Deleterious	Benign	-	3	[66]
p.T483P	c.1447A>C	Low HDL-C	DM?	Deleterious	Deleterious	Possibly Damaging	-	3	[67]
p.E490K	c.1468G>A	-	-	Deleterious	Deleterious	Possibly Damaging	1/113762 (NFE)	3	Novel
p.R496W	c.1486C>T	High HDL-C	DM?	Tolerated	Benign	Possibly Damaging	1/109 (AJ)	1	[70]
p.S499C	c.1496C>G	-	DM?	Deleterious	Deleterious	Possibly Damaging	-	3	[71]
p.L510R	c.1529T>G	Low HDL-C	DM?	Deleterious	Deleterious	Possibly Damaging	-	3	[67]
p.Y554H	c.1660T>C	Low HDL-C	DM?	Deleterious	Deleterious	Possibly Damaging	-	3	[58]
p.N567Y	c.1699A>T	Low HDL-C	DM?	Deleterious	Deleterious	Probably Damaging	-	3	[67]
p.D571G	c.1712A>G	Low HDL-C	DM	Deleterious	Deleterious	Possibly Damaging	-	3	[72]
p.D575G	c.1724A>G	Low HDL-C	DM	Tolerated	Deleterious	Probably Damaging	-	3	[73]
p.R579Q	c.1736G>A	Low HDL-C	DM	Deleterious	Deleterious	Probably Damaging	-	3	[74]
p.D585E	c.1755C>A	Low HDL-C	DM	Deleterious	Deleterious	Probably Damaging	-	3	[75]
p.V589I	c.1765G>A	Low HDL-C	DM	Tolerated	Deleterious	Benign	1/3543 (L)	3	[61]
p.G592C	c.1774G>T	Low HDL-C	DM?	Deleterious	Deleterious	Probably Damaging	1/34590 (L)	3	[67]
p.G616V	c.1847G>T	Low HDL-C	DM?	Deleterious	Deleterious	Probably Damaging	1/113758 (NFE)	4	[76]
p.Q621R	c.1862A>G	Low HDL-C	DM?	Deleterious	Deleterious	Probably Damaging	-	3	[67]
p.V628A	c.1883T>C	-	-	Deleterious	Deleterious	Probably Damaging	1/56877 (NFE)	3	Novel
p.Y712C	c.2135A>G	Low HDL-C	DM	Deleterious	Deleterious	Probably Damaging	-	3	[77]
p.V771M	c.2311G>A	-	FP	Deleterious	Benign	Benign	1/7 (L)	1	[68]
p.T774P	c.2320A>C	-	DM	Deleterious	Benign	Benign	1/293 (L)	1	[61]
p.K776N	c.2328G>C	-	DP	Tolerated	Benign	Probably Damaging	1/91 (SA)	1	[68]
p.G790D	c.2369G>A	Low HDL-C	DM	Tolerated	Deleterious	Probably Damaging	-	4	[48]
p.Y793C	c.2378A>G	-	-	Deleterious	Benign	Probably Damaging	1/15432 (NFE)	3	[26]
p.E815G	c.2444A>G	Low HDL-C	DM?	Tolerated	Benign	Benign	1/2284 (EF)	1	[70]
p.L1379F	c.4135C>T	Low HDL-C	DM	Deleterious	Deleterious	Probably Damaging	-	4	[87]
p.E1386K	c.4156G>A	Low HDL-C	DM?	Tolerated	Deleterious	Possibly Damaging	1/9196 (EA)	3	[58]

p.P1412R	c.4235C>G	Low HDL-C	DM?	Deleterious	Deleterious	Possibly Damaging	-	3	[78]
p.T1427M	c.4280C>T	-	DM	Tolerated	Benign	Benign	1/2709 (A)	2	[61]
p.C1429W	c.4287C>G	-	-	Deleterious	Deleterious	Probably Damaging	-	3	Novel
p.I1517R	c.4550T>G	Low HDL-C	DM	Deleterious	Deleterious	Probably Damaging	-	4	[79]
p.D1553H	c.4657G>C	-	-	Tolerated	Benign	Benign	1/113702 (NFE)	3	Novel
p.R1587K	c.4760G>A	-	FP	Tolerated	Benign	Benign	1/3 (All)	1	[68]
p.H1600R	c.4799A>G	Tangier	DM	Tolerated	Deleterious	Probably Damaging	-	4	[60]
p.R1615W	c.4843C>T	Low HDL-C	DM	Deleterious	Deleterious	Probably Damaging	1/32278 (NFE)	3	[72]
p.Q1826L	c.5477A>T	Low HDL-C	DM?	Tolerated	Deleterious	Possibly Damaging	-	3	[67]

*Phenotype associated with the individual variant. HDL-C: HDL cholesterol level. #Human Gene Mutation Database (HGMD) class: DM: Disease-causing mutation; DM?: Disease-causing mutation?; DP: Disease-associated phenotype; FP: *in vivo* or *in vitro* functional polymorphism; -: Not listed in HGMD. &Allele frequencies from Genome Aggregation Database (gnomAD). The highest allele frequency among the populations: African (A), Latino (L), Ashkenazi Jewish (AJ), East Asian (EA), South Asian (SA), Non-Finnish European (NFE) or European Finnish (EF) is shown. §Classification of pathogenicity was performed according to the guidelines from The American College of Medical Genetics and Genomics and The Association for Molecular Pathology (ACMG/AMP). Class 1: Not pathogenic; Class 2: Unlikely pathogenic; Class 3: Unknown pathogenicity; Class 4: Likely pathogenic; Class 5: Pathogenic. Ref.: Reference. Novel: identified at Unit for Cardiac and Cardiovascular Genetics, Oslo University Hospital.

Supplemental Table S2: Oligoes used for cloning, sequencing and mutagenesis of *ABCA1*
Oligoes for amplification of ApaI-flanking V5/his-tag in addition to cDNA primers for sequencing are listed. The forward oligo is listed for the mutagenesis primers, with mutated base underlined.

Oligo	Sequence 5'-3'
V5 his ApaI fwd	GCA TGG GCC CGA AGG TAA GCC TAT CCC TAA C
V5 his ApaI rev	GCA TGG GCC CAA CTC AAT GGT GAT GGT GAT G
cDNA ABCA1 1R	GAT ACA GGA ACC CAG AGA AG
cDNA ABCA1 2F	TTTCCGTTACCCGACTCCT
cDNA ABCA1 3F	CAGAGCGAGTACTTCGTTC
cDNA ABCA1 4F	GGAGTCTAGTCCTCTTTCCC
cDNA ABCA1 5F	GCAACAGAAGTCTGGCTCATC
cDNA ABCA1 6F	GAGGCACGGCTGAAAGAGA
cDNA ABCA1 7F	GAGAGTCCTGTGGAGGAAGA
cDNA ABCA1 8F	AATCCTGACCGGGTTGTTCCC
cDNA ABCA1 9F	ATGGATGAAGCGGACGTCCT
cDNA ABCA1 10F	CTCTCAGACCTGGGCATTTCT
cDNA ABCA1 11F	TGGCAAGTACCCCAGCCTGGAA
cDNA ABCA1 12F	CAAGATCTGGGTGAATGAG
cDNA ABCA1 13F	TTGCAATGTCCTTCGTCCCA
cDNA ABCA1 14F	CTTCCCACATTTTTGCCTGG
cDNA ABCA1 15F	CTTTGGGCTCCTGGGAGTTAA
cDNA ABCA1 16F	TGCCCTAAGTGTTGTCAAGG
K219R	GGC CTA CCA AGG GAG AAA CTG
M883I	CCA GAA GAG AAT ATC AGA AAT CTG CAT GGA GGA GG
R587W	GAG GAC ATG TGG TAC GTC TGG
W590S	ACA TGC GGT ACG TCT CGG GGG GCT T
K939M	CAA TGG AGC GGG GAT GAC GAC CAC CAT G
C1477R	AAG ATG CTG CCT GTG CGT CCC CCA GGG
Y1767D	CAG CAC AGC CGA TGT GGT GCT C
W69L	GGA ACA CTT CCT TTG GTT CAG GGG
P85L	GTT CCG TTA CCT GAC TCC TGG G
R130K	GGA CAT GCG CAA AGT TCT GAA AAC ATT ACA GC
D176N	GCT GAG GGC TAA TGT CAT TCT CC
L184S	CCA CAA GGT ATT TTC GCA AGG CTA CC
M233V	CGT TCC AAC GTG GAC ATC CTG
P250L	CAT CTC CCT TCC TGA GCA AGG AG
R282Q	GGA GTG ACA TGC AAC AGG AGG TG
E284K	GGA GTG ACA TGC GAC AGA AGG TGA TGT TTC
R306C	GCT GTG TCT TGT ATT GTC TGC GGG
S364C	AAT TTG GAG TGT AGT CCT CTT TCC CGC
P377L	GCT CTG AAG CTG CTG CTC GTT
V380I	CCG CTG CTC ATT GGG AAG ATC
D446E	GCA GGG ACA ATG AGC ACT TTT GGG
T459P	GGC TTA GAT TGG CCA GCC CAA GAC
Y482C	GGT TCT GTG TGC ACC TGG AGA G
T483P	GGT TCT GTG TAC CCC TGG AGA GAA GC
E490K	GCT TTC AAC AAG ACT AAC CAG GC
R496W	CCA GGC AAT CTG GAC CAT ATC TCG
S499C	CGG ACC ATA TGT CGC TTC ATG
L510R	CTG AAC AAG CGA GAA CCC ATA GC
Y554H	GCC CCA TCA TGT CAA GCA CAA GAT CCG A
N567Y	GTG GAG AGG ACA TAT AAA ATC AAG GAT GGG TAC TGG G
D571G	GGA CAA ATA AAA TCA AGG GTG GGT ACT GGG ACC C
D575G	GGG TAC TGG GGC CCT GGT CCT
R579Q	CCT GGT CCT CAA GCT GAC CCC
D585E	CCC TTT GAG GAA ATG CGG TAC
V589I	ATG CGG TAC ATC TGG GGG GGC
G592C	GTC TGG GGG TGC TTC GCC TAC
G616V	CGG GCA CCG AGA AGA AAA CTG TTG TCT ATA TGC

Q621R	ATG CAA CGG ATG CCC TAT CCC
V628A	CCC TAT CCC TGT TAC GCT GAT GAC ATC TTT CTG CGG
Y712C	CTG CTG CCC TGC AGT GAT CCC
V771M	CAG GAC TAC ATG GGC TTC ACA C
T774P	GTG GGC TTC CCA CTC AAG ATC
K776N	GGC TTC ACA CTC AAC ATC TTC GC
G790D	CCT GTG GCT TTT GGG TTT GAC TGT GAG TAC
Y793C	GGC TGT GAG TGC TTT GCC CTT TTT GAG G
E815G	AGT CCT GTG GGG GAA GAT GGC
L1379F	CCC CAG CCT GGA ATT TCA GCC CTG G
E1386K	GCC CTG GAT GTA CAA CAA ACA GTA CAC
P1412R	ACC AAA GAC CGT GGC TTC GGG
T1427M	ATC CCA GAC ATG CCC TGC CAG
C1429W	CAG ACA CGC CCT GGC AGG CAG GGG AGG AAG AGT GG
I1517R	GTG CAG ATC AGA GCC AAA AGC
D1553H	CCT CCG AGT CAA GAA GTT AAT CAT GCC ATC
R1587K	GGA CTG GAC ACC AAA AAT AAT GTC AAG GTG TGG
H1600R	AAG GGC TGG CGT GCA ATC AGC
R1615W	GCC ATT CTC TGG GCC AAC CTG
Q1826L	GGT GAA AAA CCT GGC AAT GGC TG

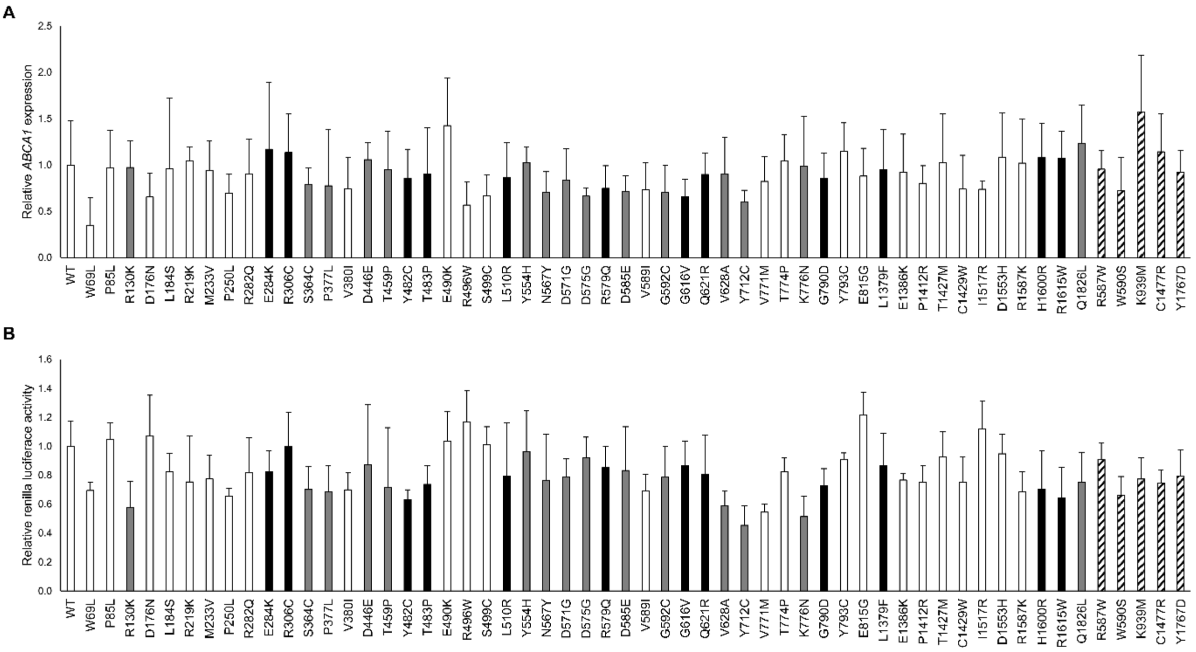

Supplemental Figure S1: **Transient transfection efficiency.** Expression and functionality differences due to transient transfection efficiencies was minimized by monitoring general mRNA expression of overexpressed *ABCA1* and luminescence from co-transfected renilla in each sample in the cholesterol efflux assay. A) cDNA was synthesized from RNA isolated from transiently transfected HEK293 cells, and analyzed using PrimeTime Predesigned qPCR Assay primers (Integrated DNA Technologies, Coralville, IA) for ABCA1 (Hs.PT.58.27452429) and glyceraldehyde-3-phosphate dehydrogenase (GAPDH) (Hs.PT.39a.22214836). Amount of target mRNA was determined and normalized to the housekeeping gene GAPDH using the $2^{-\Delta\Delta Ct}$ method [57], and is presented as mean of four independent experiments. B) Intra-assay transfection differences in the cholesterol efflux assay was monitored by co-transfection of each *ABCA1* variant with a renilla luciferase plasmid, phRL (Promega, Madison, WI) at a ratio of 9:1. The luminescence was measured using Renilla Luciferase Assay System (Promega) and read on a Synergy H1 Plate Reader (BioTek, Winooski, VT), and is presented as mean of the four different experiments presented in Fig. 1 in the main manuscript.

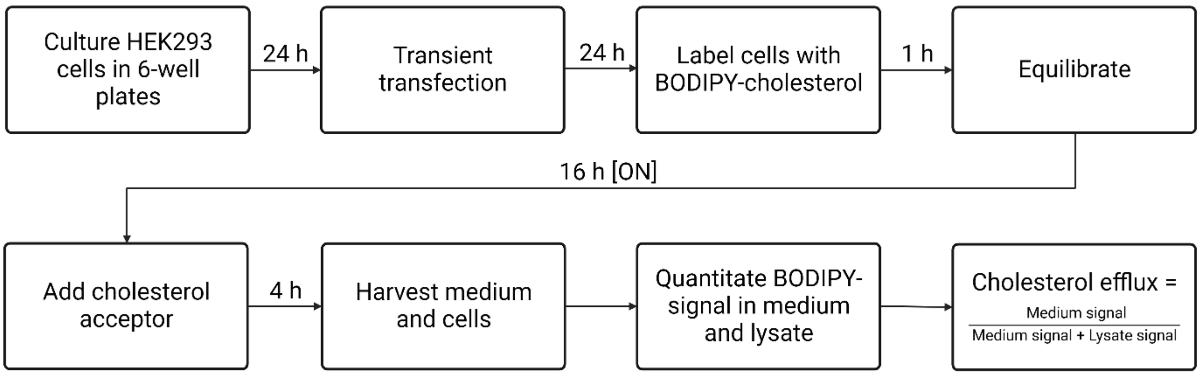

Supplemental Figure S2: **Cholesterol efflux assay.** The general work flow was adapted from other cholesterol efflux studies [23, 24, 32, 33]. Optimization of individual steps in the workflow are presented in Suppl. Fig. S3-S6. Created with BioRender.com.

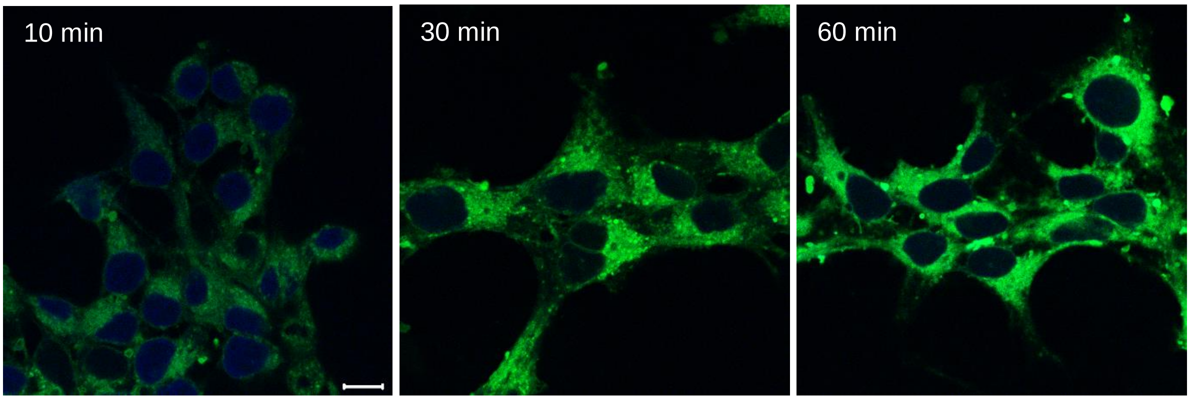

Supplemental Figure S3: **BODIPY-cholesterol labeling of HEK293 cells.** Untransfected cells were incubated with BODIPY-cholesterol (green; Cayman Chemicals, Ann Arbour, MI) for 10, 30 and 60 min and assessed using confocal microscopy after equilibration. 4′,6-diamidino-2-phenylindole (DAPI, blue; Invitrogen, Carlsbad, CA) was used as nuclear staining. BODIPY-cholesterol was diluted in ethanol and cell medium concentration was kept <1% (v/v) [80]. A scale bar of 10 µm is shown. Highest fluorescence signal was obtained at 60 min, which was used in the optimized protocol.

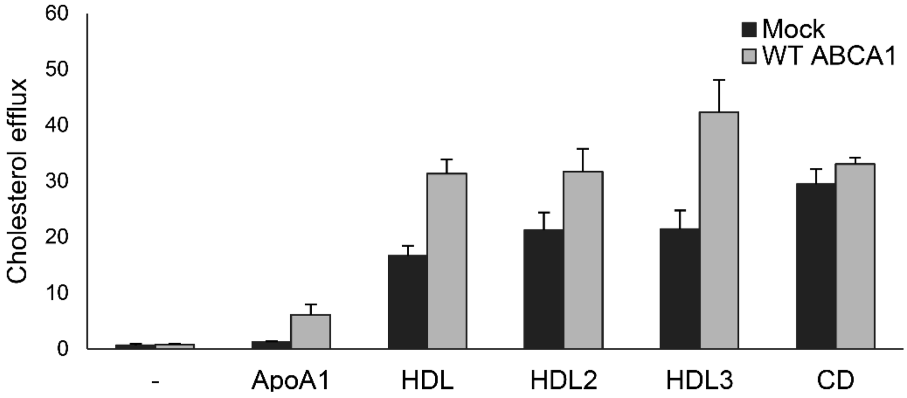

Supplemental Figure S4: Evaluation of cholesterol acceptors. Cholesterol efflux from BODIPY-loaded and equilibrated transiently transfected HEK293 cells was determined after incubation with 10 µg/ml recombinant ApoA1 (ab50239, Abcam, Cambridge, UK), 100 µg/ml HDL (ρ = 1.063-1.210 g/ml), HDL_2 (ρ = 1.090-1.125 g/ml) or HDL_3 (ρ = 1.125-1.210 g/ml) (n = 3, error bar represents 1 SD). Methyl-β-cyclodextrin (CD; Sigma-Aldrich, St Louis, MO) 5 mM was used as a positive control. Crude HDL was selected as preferred cholesterol acceptor in the optimized protocol due to low cost compared to ApoA1 and attainability compared to HDL_3.

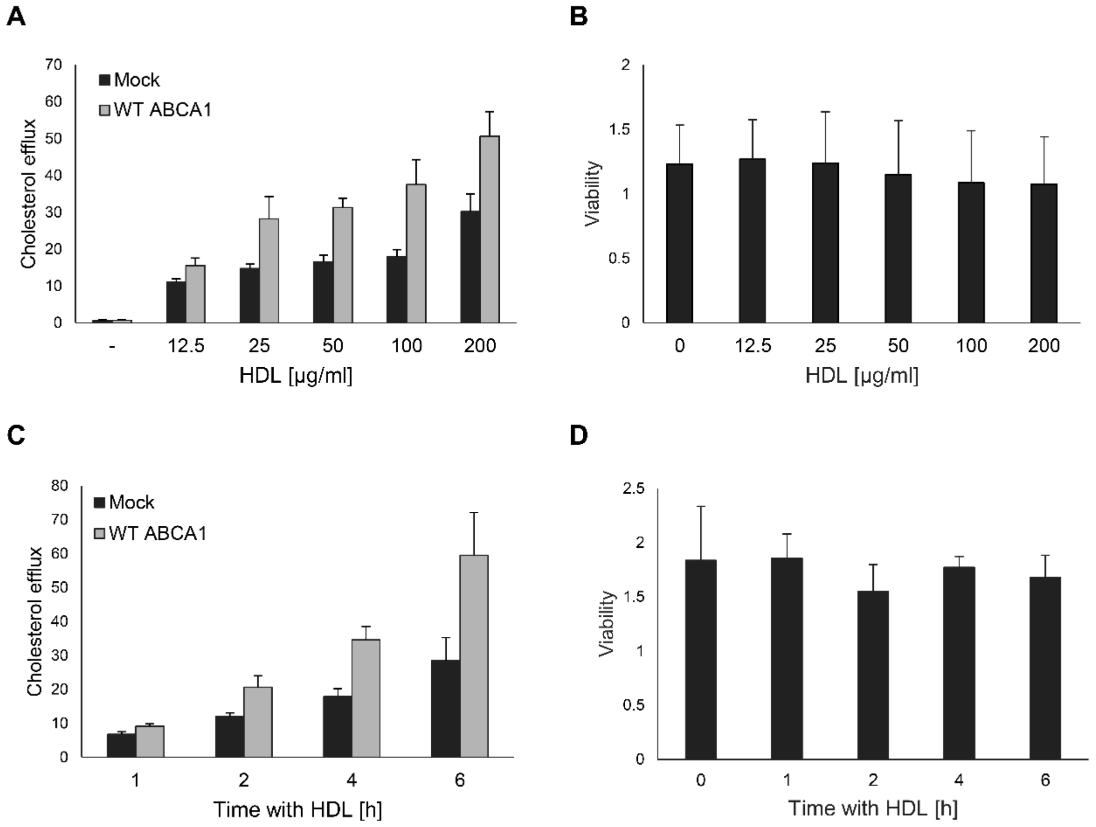

Supplemental Figure S5: Evaluation of acceptor incubation time and concentration. Cholesterol efflux from BODIPY-loaded and equilibrated transiently transfected HEK293 cells was measured, showing A) incubation with HDL at different concentrations for 4 h, and C) different incubation times using 100 µg/ml HDL (n = 3, error bar represents 1 SD). B+D) Cell viability was monitored using an MTT assay (ab211091; Abcam) in accordance with the manufacturer's instructions (n =3, error bar represents 1 SD). An acceptor incubation time of 4 h and a concentration of 100 µg/ml was used in the optimized protocol.

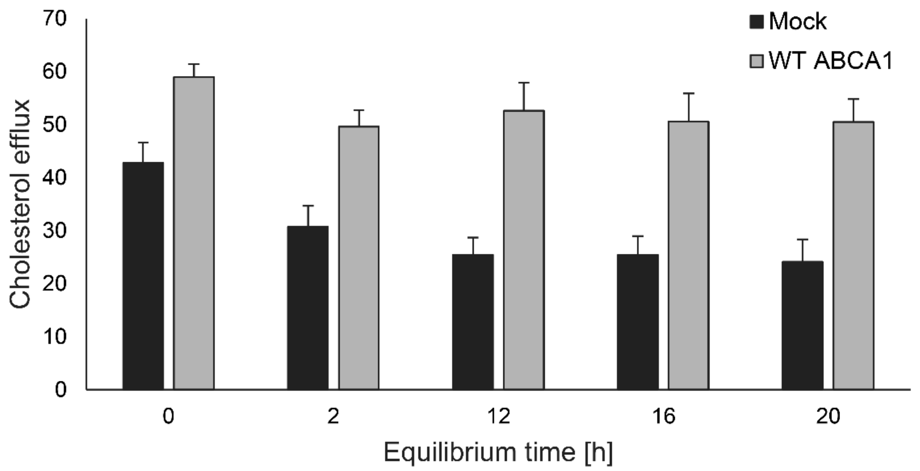

Supplemental Figure S6: Evaluation of equilibrium times. Transiently transfected and BODIPY-loaded HEK293 cells were equilibrated for the indicated times. No cholesterol leakage was detected in the equilibrium medium (data not shown). After subsequent incubation with 100 µg/ml HDL for 4 h, cholesterol efflux was determined (n = 3, error bar represents 1 SD). The equilibrium time was set to 16 h in the optimized protocol.

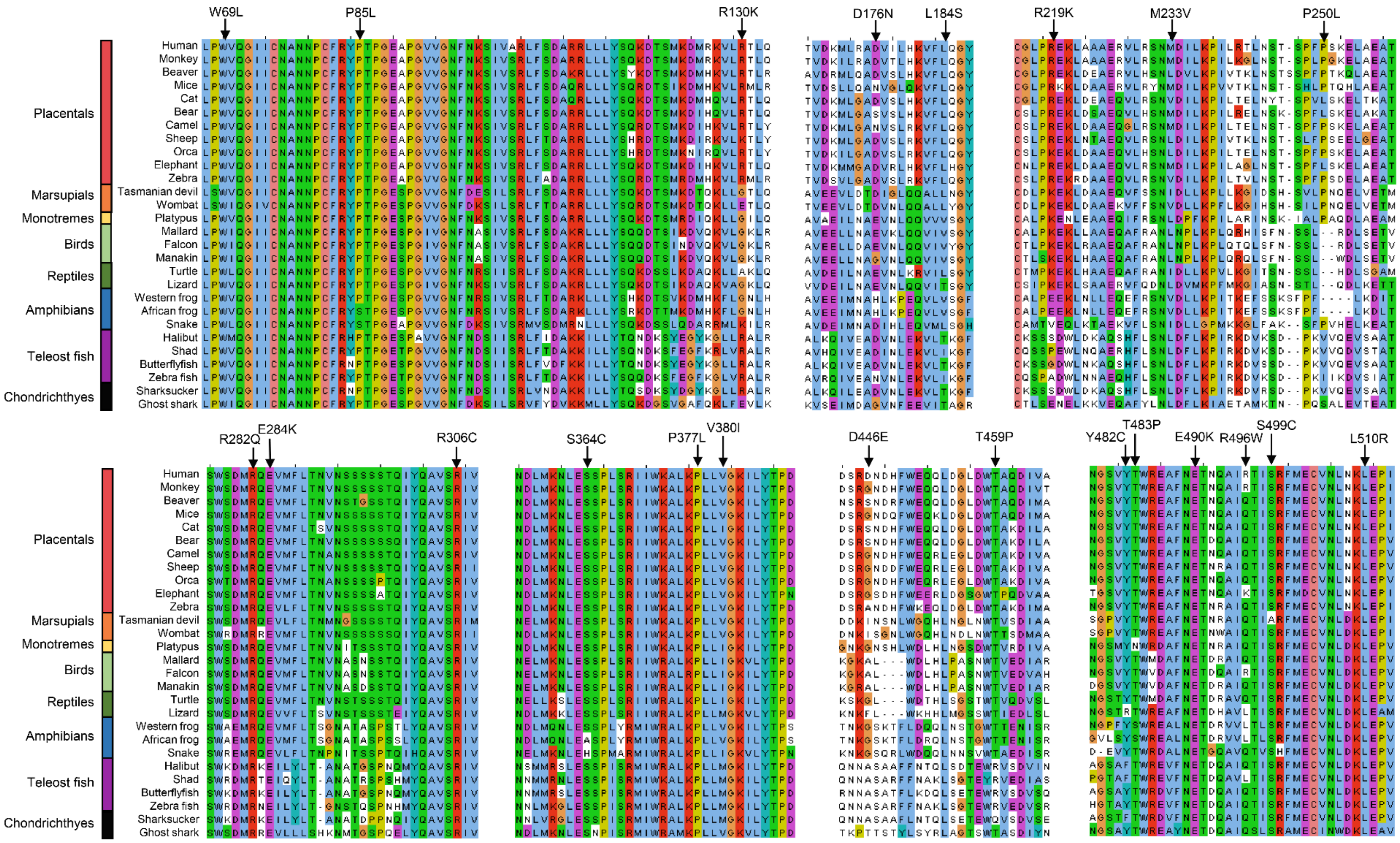
W69L
P85L
R130K
D176N
L184S
R219K
M233V
P250L
R282Q
E284K
R306C
S364C
P377L
V380I
D446E
T459P
Y482C
T483P
E490K
R496W
S499C
L510R
Placentals
Marsupials
Monotremes
Birds
Reptiles
Amphibians
Teleost fish
Chondrichthyes
Human
Monkey
Beaver
Mice
Cat
Bear
Camel
Sheep
Orca
Elephant
Zebra
Tasmanian devil
Wombat
Platypus
Mallard
Falcon
Manakin
Turtle
Lizard
Western frog
African frog
Snake
Halibut
Shad
Butterflyfish
Zebra fish
Sharksucker
Ghost shark

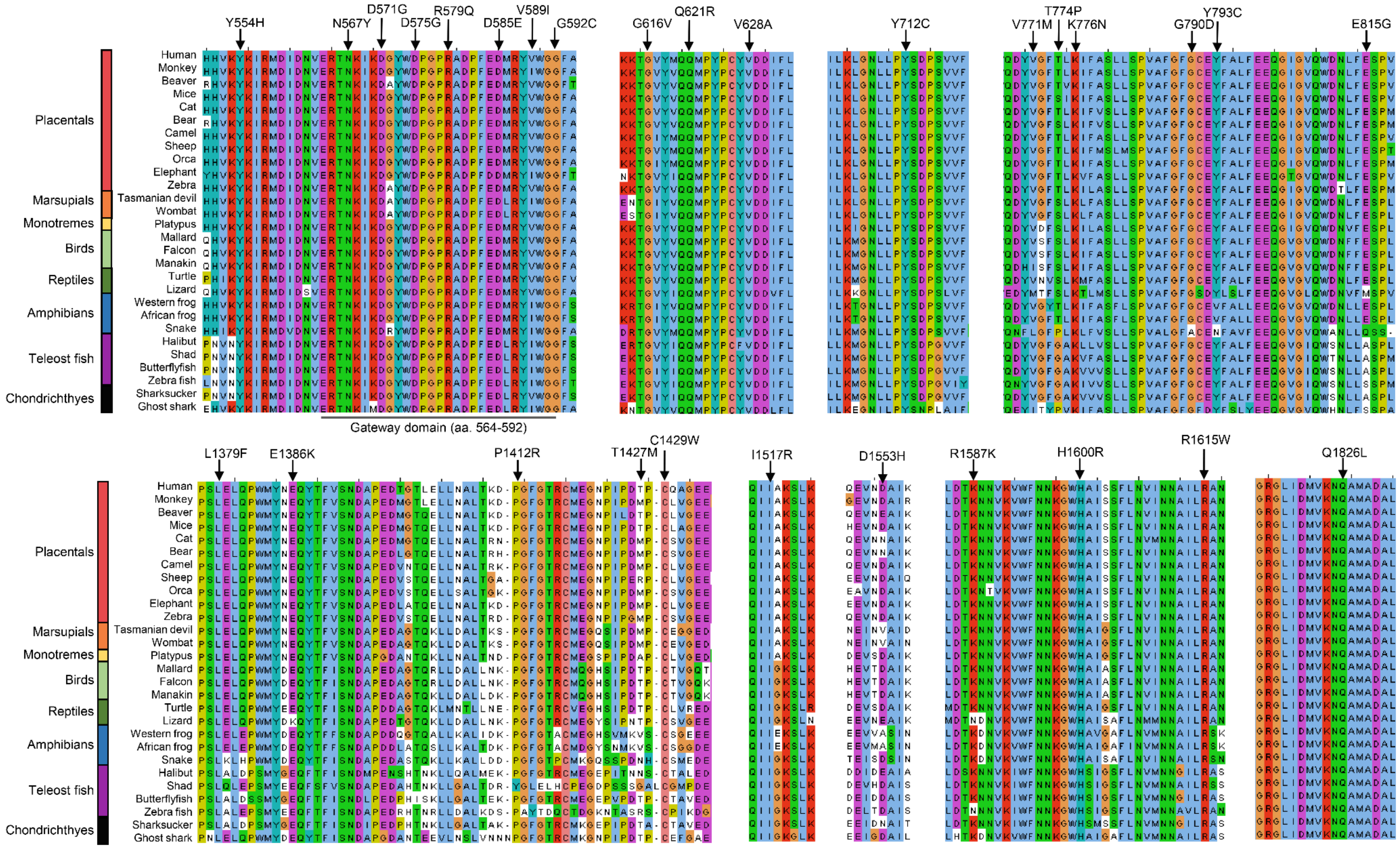
Y554H
N567Y
D571G
D575G
R579Q
D585E
V589I
G592C
G616V
Q621R
V628A
Y712C
V771M
T774P
K776N
G790D
Y793C
E815G
Gateway domain (aa. 564-592)
L1379F
E1386K
P1412R
T1427M
C1429W
I1517R
D1553H
R1587K
H1600R
R1615W
Q1826L
Placentals
Marsupials
Monotremes
Birds
Reptiles
Amphibians
Teleost fish
Chondrichthyes
Human
Monkey
Beaver
Mice
Cat
Bear
Camel
Sheep
Orca
Elephant
Zebra
Tasmanian devil
Wombat
Platypus
Mallard
Falcon
Manakin
Turtle
Lizard
Western frog
African frog
Snake
Halibut
Shad
Butterflyfish
Zebra fish
Sharksucker
Ghost shark

Supplemental Figure S7: Multiple sequence alignment of 51 missense variants in *ABCA1*. Protein sequences that are homologous to human ABCA1 were obtained from the NCBI RefSeq database resources [81] by employing standard BLAST sequence searching [82]. Only sequences with sequence identity above 50% of that of human *ABCA1* were included in the dataset. UniProt database [83] was used to evaluate and ensure good quality of the selected sequences. Multiple sequence alignments were generated in Jalview [84] with the use of MUSCLE [85], and visualized with the Clustal coloring scheme. The gateway domain identified by Segrest et al. [12] is indicated. The species studied were: Human, *Saimiri boliviensis boliviensis* (Black-capped squirrel monkey), *Castor canadensis* (North American beaver), *Mus musculus* (House mouse), *Felis catus* (Cat), *Ursus arctos* (Brown bear), *Camelus bactrianus* (Bactrian camel), *Ovis aries* (Sheep), *Orcinus orca* (Orca or killer whale), *Elephas maximus indicus* (Indian elephant), *Sarcophilus harrisii* (Tasmanian devil), *Vombatus ursinus* (Common wombat), *Ornithorhynchus anatinus* (Platypus), *Anas platyrhynchos* (Mallard), *Falco naumanni* (Fallcon), *Manacus candei* (White-collared manakin), *Mauremys reevesii* (Chinese pond turtle), *Lacerta agilis* (Sand lizard), *Xenopus tropicalis* (Western clawed frog), *Xenopus laevis* (African clawed frog), *Geotrypetes seraphini* (Gaboon caecilian), *Hippoglossus stenolepis* (Pacific halibut), *Alosa sapidissima* (American shad), *Chelmon rostratus* (Copperband butterflyfish), *Danio rerio* (Zebra fish), *Echeneis naucrates* (Live sharksucker) and *Callorhinchus milii* (Australian ghostshark).

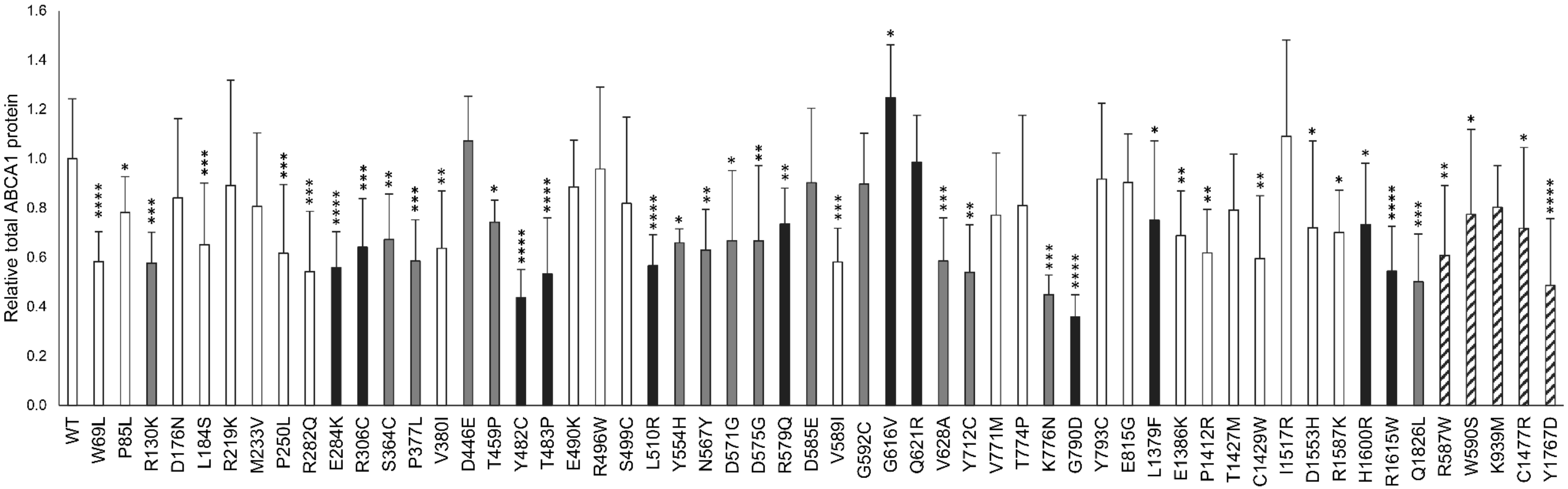

Supplemental Figure S8: Relative total protein for *ABCA1* missense variants. Relative total protein levels of missense variants (solid) and 5 loss-of-function control variants (striped) normalized to WT ABCA1 presented as mean of four independent experiments. Error bar represents 1 SD. **P* < 0.05, ***P* < 0.01, ****P* < 0.001, *****P* < 0.0001, two-tailed *t*-test vs WT ABCA1. ABCA1 was detected using a V5-HRP antibody (#R961-25, Invitrogen) and corrected for loading control β-actin for each sample. Coloration corresponds to variant assessment from cholesterol efflux assay presented in Fig. 1A in main manuscript (white: benevolent; grey: variant of uncertain significance; black: loss-of-function).

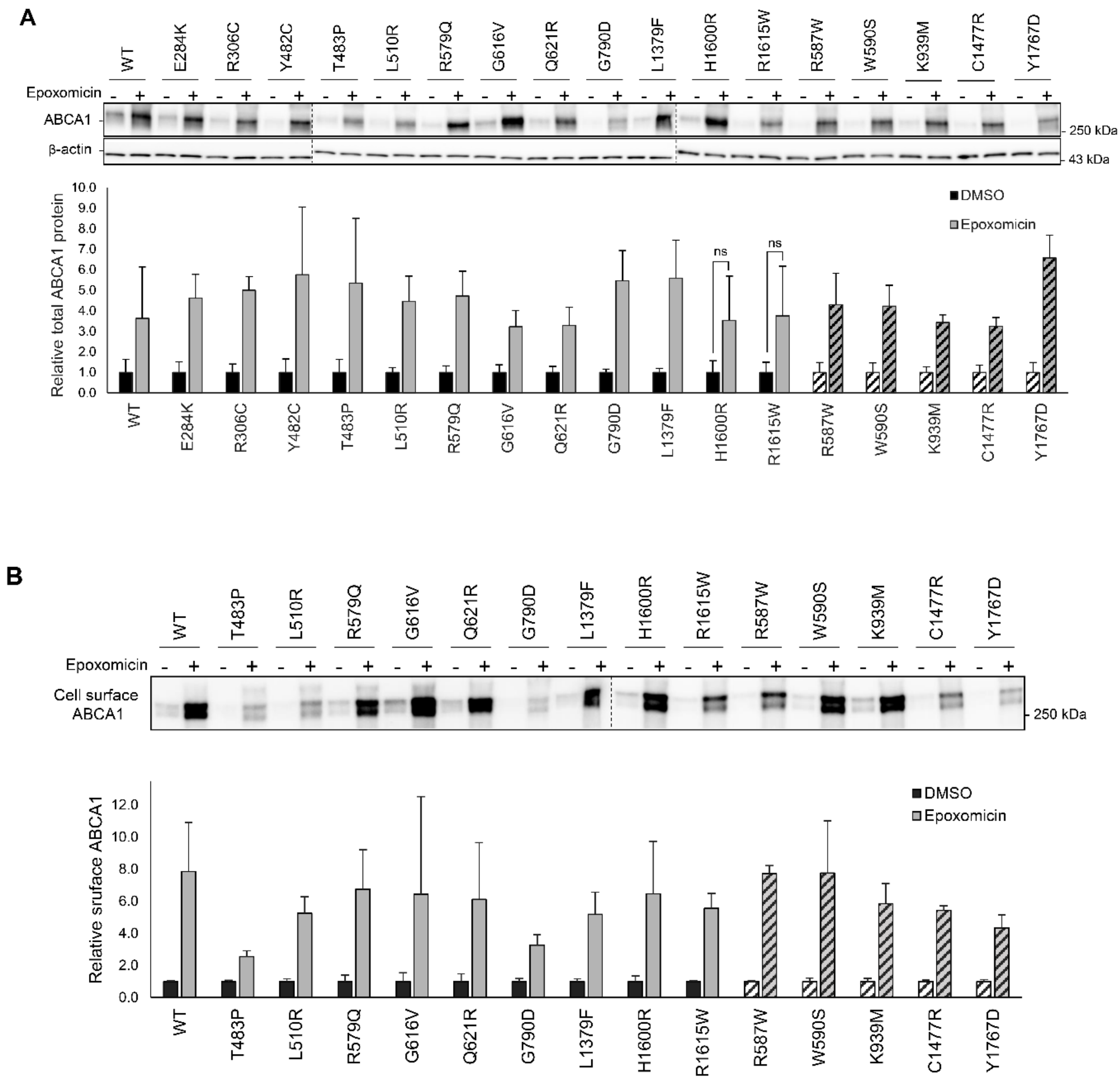

Supplemental Figure S9: Total and cell surface located ABCA1 after epoxomicin treatment. A) After epoxomicin treatment (1 µM, 20 h), the total ABCA1 protein levels in transiently transfected HEK293 cells were assessed by western blot analysis. One representative blot is shown and relative total protein levels of loss-of-function variants (solid) and five control variants (black striped) normalized to vehicle-treated cells presented as mean of three independent experiments. Error bar represents 1 SD. $P < 0.05$, one-tailed *t*-test vs vehicle-treated cells. Non-significant expression difference (ns) is indicated. ABCA1 was detected using a V5-HRP antibody (Invitrogen) and corrected for loading control β-actin for each sample. B) Cell surface located ABCA1 was analyzed for the variants that did not regain functionality after epoxomicin treatment (1 µM, 20 h). Transiently transfected HEK293 cells were biotinylated and equal amounts of lysate was immunoprecipitated using Dynabeads™ MyOne™ Streptavidin T1 (Invitrogen). One representative blot is shown and relative cell surface expression of loss-of-function variants (solid) and five control variants (black striped) normalized to vehicle-treated cells presented as mean of three independent experiments. Error bar represents 1 SD. $P < 0.05$, one-tailed *t*-test vs vehicle-treated cells. ABCA1 was detected using a V5-HRP antibody. Dotted lines denote where blots have been merged.

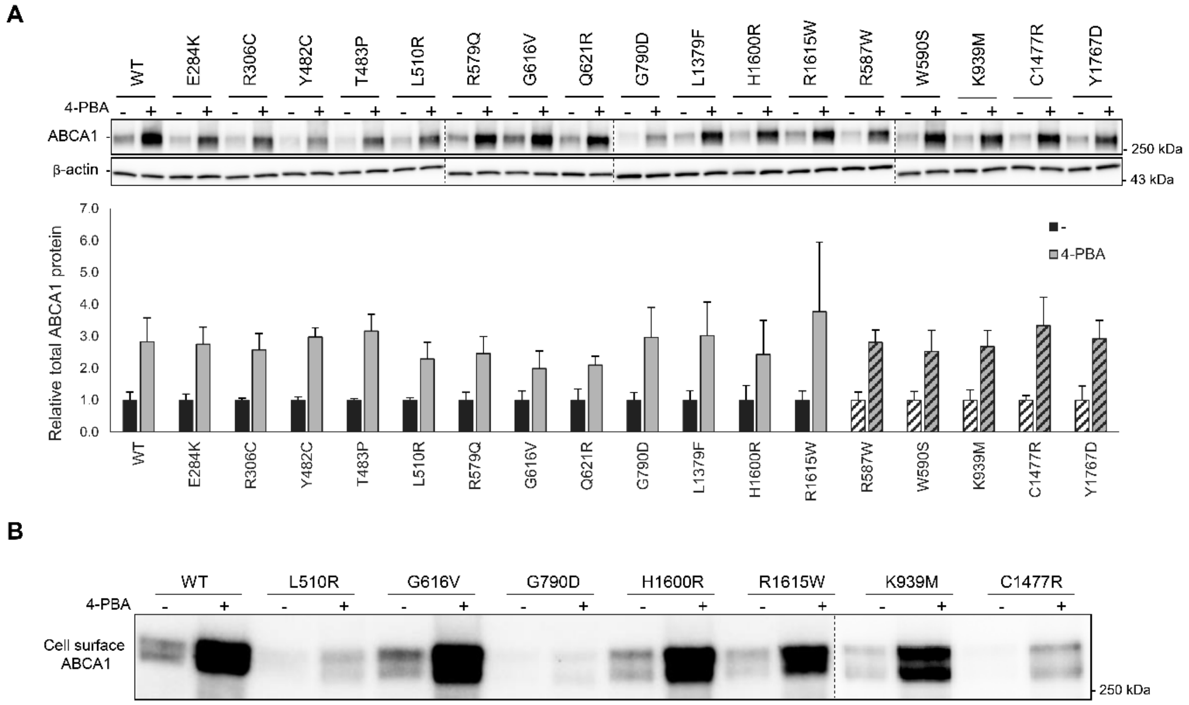

Supplemental Figure S10: Total and cell surface located ABCA1 after 4-PBA treatment. A) After 4-PBA treatment (10 mM, 20 h), the total ABCA1 protein levels in transiently transfected HEK293 cells were assessed by western blot analysis. One representative blot is shown and relative total protein levels of loss-of-function variants (solid) and five control variants (black striped) normalized to vehicle-treated cells are presented as mean of three independent experiments. Error bar represents 1 SD. $P < 0.05$, one-tailed *t*-test vs vehicle-treated cells. ABCA1 was detected using a V5-HRP antibody (Invitrogen) and corrected for loading control β-actin for each sample. B) Cell surface located ABCA1 was analyzed for the five loss-of-function variants that did not regain functionality after 4-PBA treatment (10 mM, 20 h). Transiently transfected HEK293 cells were biotinylated and equal amounts of lysate was immunoprecipitated using Dynabeads™ MyOne™ Streptavidin T1 (Invitrogen). ABCA1 was detected using a V5-HRP antibody. One representative blot is shown (n = 3) and relative cell surface expression is presented in Fig 4D in the main manuscript. Dotted lines denote where blots have been merged.

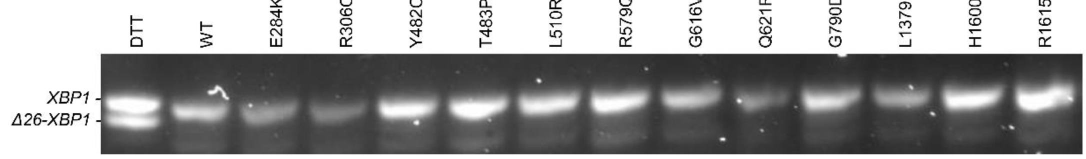

Supplemental Figure S11: Analysis of ER stress caused by *ABCA1* variants. ER stress activates phosphorylation of inositol-requiring enzyme 1 that activates its endoribonuclease activity and causes skipping of 26 nucleotides in mRNA of X-box binding protein 1 (Δ26-*XBP1*) [86]. *XBP1* mRNA from HEK293 cells transiently transfected with the 12 *ABCA1* loss-of-function variants was amplified using the forward primer: 5'-CTGGAAAGCAAGTGGTAGA-3' and the reverse primer: 5'-CTGGGTCCTTCTGGGTAGAC-3', and analyzed using RT-PCR and gel electrophoresis (n = 3). Dithiothreitol (DTT; 5 mM; Sigma-Aldrich) was used as positive control. There was no apparent difference in the degree of the spliced *XBP1* fragment between the WT ABCA1 and the loss-of-function variants.

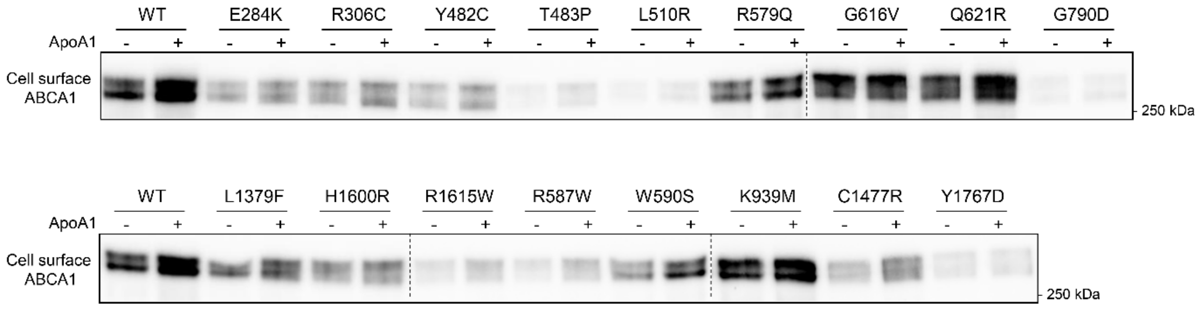

Supplemental Figure S12: Stabilization of ABCA1 loss-of-function variants by recombinant ApoA1. Surface-exposed ABCA1 of transiently transfected HEK293 cells after ApoA1 treatment (10 µg/ml, 2 h) was biotinylated and assessed by western blot analysis. Equal amounts of total lysate was immunoprecipitated using Dynabeads™ MyOne™ Streptavidin T1 (Invitrogen) and ABCA1 was detected using an anti-V5-HRP antibody (Invitrogen). One representative blot is shown (n = 3) and relative cell surface expression is presented in Fig 5 in the main manuscript. Dotted lines denote where blots have been merged.

Supplemental Table S3: Clinical parameters for the 12 *ABCA1* loss-of-function variants. Gender, zygosity and serum levels of total cholesterol (mmol/l), HDL cholesterol (mmol/l) and ApoA1 (mg/dl) reported for subjects with loss-of-function variants in *ABCA1*. The additional *ABCA1* variant is indicated for the compound heterozygous subjects. Also shown is normal range for HDL cholesterol and ApoA1.

Variant	Gender	Zygosity	Total cholesterol	HDL-C	ApoA1	Reference
p.E284K	F	He	7.11	0.2	30	[66]
p.R306Y	M	He	3.05	0.3	67	[67]
p.Y482C	F	c.He (N1800H)	4.5	0.4	57	[66]
p.T483P	F	c.He (C1477F)	2.66	0.13	30	[67]
p.L510R	M	He	3.78	0.4	76	[67]
p.R579Q	-*	c.He (V771M)	4.35[§]	0.84[§]	106[§]	[74]
p.G616V	M	He	3.04	0.39	32	[76]
p.Q621R	M	He	4.68	0.36	64	[67]
p.G790D	M	c.He (N1800H)	2.66	0.18	27	[48]
p.L1379F	F	c.He (V1704D)	3.8	<0.1	<30	[87]
p.H1600R	M	Ho	1.24	0.05	0	[53]
p.R1615W	-	He	-	$<10^{th}$ centile	-	[72]
Normal range[#]	M	-	-	0.8-2.1	100-200	-
	F	-	-	1.0-2.7	110-230	-

HDL-C: HDL cholesterol; M: male; F: female; He: heterozygous; Ho: homozygous; c.He: compound heterozygous. *gender not specified. [§]mean values of 36 *ABCA1* variant carriers. [#]values from Department of Medical Biochemistry, Oslo University Hospital, Norway.
